# Uncertainty in hydrological analysis of climate change: multi-parameter vs. multi-GCM ensemble predictions

**DOI:** 10.1038/s41598-019-41334-7

**Published:** 2019-03-21

**Authors:** Younggu Her, Seung-Hwan Yoo, Jaepil Cho, Syewoon Hwang, Jaehak Jeong, Chounghyun Seong

**Affiliations:** 1 Department of Agricultural and Biological Engineering/Tropical Research and Education Center, Institute of Food and Agricultural Sciences, University of Florida, Homestead, Florida USA; 2 Department of Rural and Bio-Systems Engineering, Chonnam National University, Gwangju, Republic of Korea; 3 Climate Services and Research Department, APEC Climate Center, Busan, Republic of Korea; 4 Department of Agricultural Engineering, Institute of Agriculture and Life Science, Gyeongsang National University, Jinju, Republic of Korea; 5 Texas A&M AgriLife Research, Texas A&M University, Temple, Texas United States; 6 Bureau of Watershed Management & Modeling, St. Johns River Water Management District, Palatka, Florida USA

## Abstract

The quantification of uncertainty in the ensemble-based predictions of climate change and the corresponding hydrological impact is necessary for the development of robust climate adaptation plans. Although the equifinality of hydrological modeling has been discussed for a long time, its influence on the hydrological analysis of climate change has not been studied enough to provide a definite idea about the relative contributions of uncertainty contained in both multiple general circulation models (GCMs) and multi-parameter ensembles to hydrological projections. This study demonstrated that the impact of multi-GCM ensemble uncertainty on direct runoff projections for headwater watersheds could be an order of magnitude larger than that of multi-parameter ensemble uncertainty. The finding suggests that the selection of appropriate GCMs should be much more emphasized than that of a parameter set among behavioral ones. When projecting soil moisture and groundwater, on the other hand, the hydrological modeling equifinality was more influential than the multi-GCM ensemble uncertainty. Overall, the uncertainty of GCM projections was dominant for relatively rapid hydrological components while the uncertainty of hydrological model parameterization was more significant for slow components. In addition, uncertainty in hydrological projections was much more closely associated with uncertainty in the ensemble projections of precipitation than temperature, indicating a need to pay closer attention to precipitation data for improved modeling reliability. Uncertainty in hydrological component ensemble projections showed unique responses to uncertainty in the precipitation and temperature ensembles.

## Introduction

General circulation models (GCMs) have been developed by many national and international research institutions and agencies and served as useful, and probably the only kind of tools to predict future climate^1,2^. Since each GCM has been developed based on its own assumptions and unique mathematical representations of physical climate system processes, different climate projections are provided^3^. Thus, climate model selection is not only a watershed modeler’s first decision in a hydrological analysis of climate change, but it is also one of the most critical ones. However, the selection is often undertaken with limited information regarding quality and reliability^1^.

The Intergovernmental Panel on Climate Change (IPCC) launched the Coupled Model Intercomparison Project Phase 5 (CMIP5) in the fifth Assessment Report (AR5), whereby a multi-GCM ensemble analysis was facilitated through the provision of climate model outputs that comply with community standards^4,5^. Multi-GCM ensembles have served as a framework for accommodating probabilistic approaches in interpreting climate predictions and developing climate adaptation plans, and many studies have attempted to quantify uncertainty with the information of ensemble spread and to identify its sources^1,6–11^. Ensemble averaging can improve the accuracy of a climate projection by allowing GCM errors to cancel each other out and GCMs that poorly performed to be downweighted^12^. However, the approach often does not employ all models available and thus may underestimate uncertainty and/or produce a bias in an ensemble prediction^8,13^. Further, the interpretation of an ensemble averaging prediction remains challenging due to “the lack of consensus on models”^14,15^.

Because of the global nature of the climate system and the complexity of the underlying climate physics, climate change impact assessments are often implemented in continental and regional extents, which, however, are not the scales at which most hydrological analyses and water resources management are carried out^6,16–20^. A large-scale analysis does not consider detailed hydrological processes, and localized impacts may not be efficiently represented at such scale^18,21,22^. For instance, hillslope processes including infiltration and overland flow transport are more dominant and influential in the hydrology and ecosystem of a small watershed, while channel routing and groundwater flow are processes controlling the overall hydrological response of a large watershed^23–25^. In addition, it is reasonable to assume a homogeneous landscape for a hillslope, whereas a large-scale watershed tends to have considerable heterogeneity in its landscape^16^. The responses of individual spatial units of a large watershed are likely to be intermingled with each other and dampened through prolonged overland and channel processes^25–27^. The hydrological responses of local headwater watersheds to climate would, therefore, be clearly explained at small spatial scales.

Many different hydrological models, from lumped to distributed, have been utilized in climate change studies: the variable infiltration capacity (VIC) model^7,28^, Hydrologiska Byråns Vattenbalansavdelning (HBV) model^9,29^, Water - Global Analysis and Prognosis (WaterGAP) model^30,31^, Lund-Potsdam-Jena managed Land (LPJmL) model^32,33^, and the Soil and Water Assessment Tool (SWAT) model^34,35^, as well as simple models such as ABCD and Budyko^36–40^. Complicated models can simulate detailed hydrological processes, but the sizable input data and parameter requirements tend to result in uncertainty and inefficiency^41^. The level of model complexity required would increase with decreases in the spatiotemporal resolutions at which hydrological processes are simulated and with increases in the number of hydrological processes to be simulated^42^. When model predictions are found to be inaccurate, a modeler may want to increase the complexity of the model^43^. However, the complexity of a model to be used should be balanced with the number of available observations, measurements, and information that can constrain the behavior of the model^44,45^. For instance, a study demonstrated that only five parameters would be enough to represent rainfall-runoff conversion processes when an appropriate level of model sensitivity and low correlation between parameters are ensured^46^. Simpler models are therefore preferable as long as they can predict hydrological variables and components of interest at the required levels of accuracy and detail, especially when the overall far future hydrological responses of a watershed are of interest.

An understanding of the sources and influences of uncertainty helps to identify ways to efficiently improve the robustness and reliability of a climate change impact analysis, whereby the subsequent development of climate change impact mitigation strategies and water resource management plans can be more effective^47^. Equifinality is a concept in which there are multiple parameter sets providing equally good or acceptable model outputs. Equifinality is one of the main sources of uncertainty in hydrological modeling, and many methods have been proposed to quantify equifinality and the resulting uncertainty^48^. While it is known that equifinality decreases with increases in the number of observations and decreases in the number of calibration parameters, equifinality is inevitable, and its impact is substantial in hydrological modeling^41^.

There are only a few known studies about the influence of equifinality of hydrological models on climate change impact assessment. A study argued that uncertainty originating from hydrological models is as large as that of climate models^49^. Another study demonstrated that hydrological model structure uncertainty is more influential than parameter uncertainty in the assessment of climate change impact on a snow-dominated river basin^50^. It was found that a climate change impact assessment could be significantly affected by hydrological model selection and parameter calibration^51^. Several studies showed that the selection of a hydrological model (structural uncertainty) could be much more critical than GCM selection in the hydrological analysis of climate change^52–54^. On the other hands, other studies demonstrated that hydrological model parameter uncertainty is the least influential; notably, though, the numbers of parameter sets incorporated into their studies were small (10 to 20), indicating there was a strong possibility that the impacts of equifinality might be underestimated^55,56^.

This study compared the significance of selections of GCMs and hydrological model parameters (equifinality) on hydrological assessment of climate change for local headwater watersheds. For this comparison to be meaningful, we quantified uncertainty in multi-GCM and multi-parameter ensemble projections made for the weather and hydrology of multiple watersheds. In addition, 35 ensemble members including 22 CMIP5 GCMs and their variants (hereafter 35 GCMs) were considered in this study, and 61 headwater watersheds found in the Ohio River basin were incorporated to show variability in quantified uncertainty across different watersheds and climate. A monthly water balance model, ABCD, was employed as a mathematical representation of the mechanisms that control the responses of the hydrological components to climate variability. The behavioral parameter sets of the water balance model that were developed for each watershed were identified using the Generalized Likelihood Uncertainty Estimator (GLUE) framework^57^, and multiple acceptability thresholds were applied to see the sensitivity of quantified equifinality impacts on the overall uncertainty to the subjective threshold selections. This study also showed how uncertainty in temperature and precipitation projections influence hydrological variables including direct runoff, soil water content, evapotranspiration and groundwater at the local watershed scale.

## Results

### Projected precipitation and temperature

**Figure 1:**
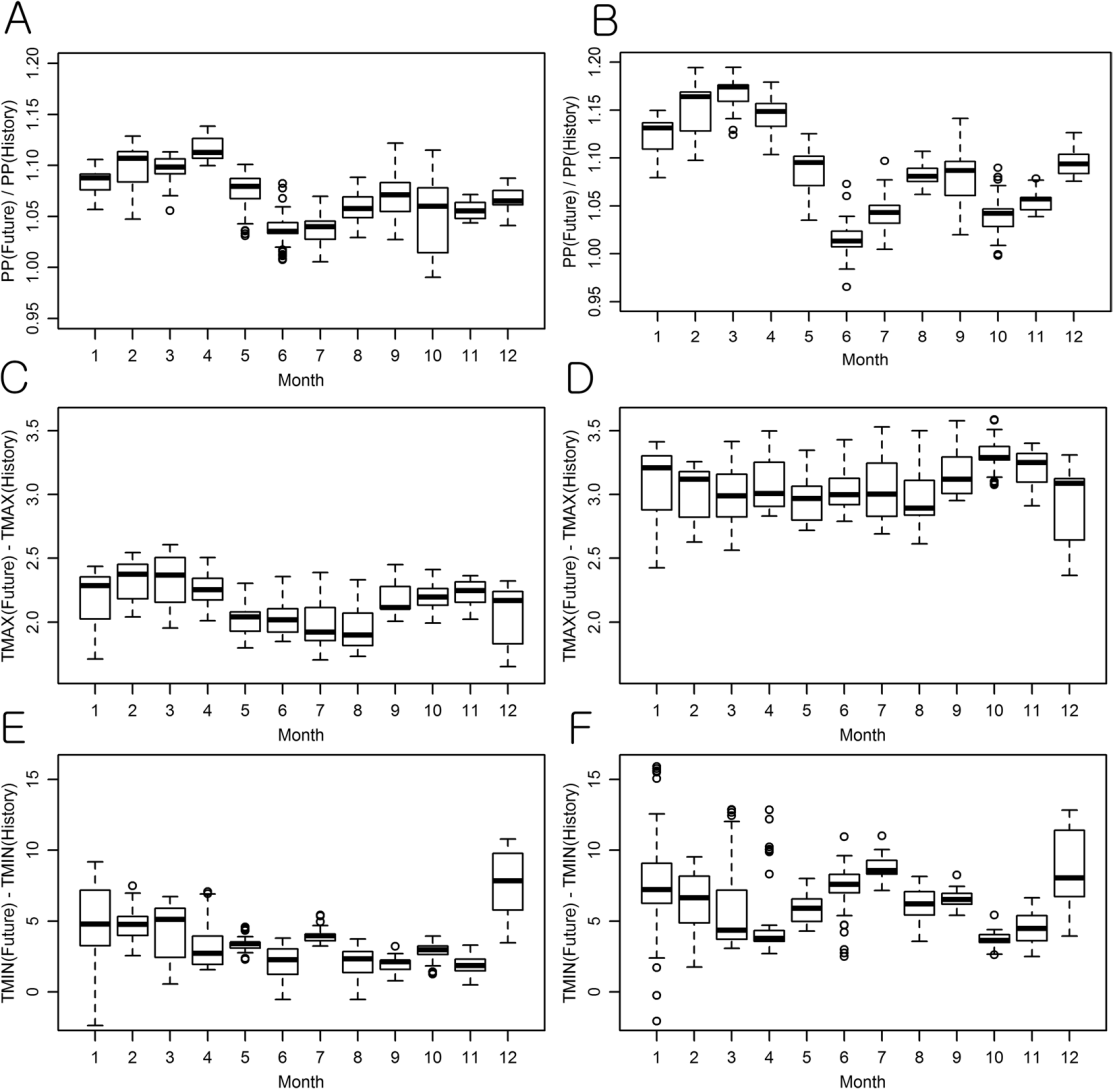
Overall monthly variations in the projected changes of precipitation (projected/historical) and temperature (projected - historical) across the study watersheds. (**A,B**): precipitation (ratio); (**C,D**): maximum temperature (°C); (**E,F**): minimum temperature (°C); (**A,C,E**): RCP 4.5; (**B,D,F**): RCP 8.5.

The precipitation and temperature projections made by the 35 GCMs were averaged by the months and by the study watersheds to investigate the overall trends of future climate in the Ohio River basin (Fig. 1). The annual average precipitation of the Ohio River watersheds was projected to increase by 6.8% and 8.8% from 2020 to 2099 under the Representative Concentration Pathway (RCP) 4.5 and RCP 8.5 scenarios, respectively. The projected monthly precipitations showed large seasonal variations, with up to 14% and 19% increases under the RCP 4.5 and RCP 8.5 scenarios, respectively (Fig. 1(A,B)). The increased rates were higher in winter and spring than in summer, which is in agreement with findings reported in the literature^58^.

The annual temperature was projected to increase by 2.2 °C and 3.6 °C on average in the watersheds under the RCP 4.5 and RCP 8.5 scenarios, respectively, compared to the historical average temperature of 12 °C (Table 1), which is also consistent with the previous study^58^. The monthly maximum and minimum temperatures were predicted to increase in most of the watersheds. The variations of the minimum temperature across the study watersheds (variations in the minimum temperature projections by the watersheds or the heights of the boxes in the boxplots, Fig. 1) were more significant than those of the maximum temperature. The amount of variation in the maximum temperature across the watersheds was relatively consistent over months, but the minimum temperature widely varied by the watersheds during winter, indicating that the climate change of the Ohio River watersheds would be more evident in terms of the minimum (rather than maximum) temperature. The projected increases of the minimum temperature were mapped to identify spatial trends in the projections (Fig. S1). In the maps prepared for November, December, and January, the differences between the historical and projected temperatures increased across the basin from the southwest (mild temperate or humid subtropical) to the northeast (humid continental). The spatial variability was higher in January than November and December, and it was greater in the RCP 8.5 scenario than the RCP 4.5. According to the Köppen-Geiger climate classification system^59^, the Ohio River basin lies on four climate zones, Cfa, Cfb, Dfa, and Dfb, where “C” signifies “Temperate”, “f” represents “Without Dry Season”, “a” means “Hot Summer”, “b” means “Warm Summer”, and “D” signifies “Cold” (Fig. S2). The comparison between the spatial patterns of projected temperature increases and the climate classification map suggested that the winter hydrology of areas that have relatively cold weather (e.g. Dfb in the northeast) can be more substantially influenced by projected temperature changes, compared to temperate areas (e.g. Cfa in the southwest).

**Table 1:** Overall changes of the climate variables and hydrologic components projected by the multi-GCM, multi-parameter, and multi-watershed ensembles.

Variables	Statistics	Historical	RCP 4.5	RCP 8.5
Temperature (TAV)	Average (°C)	12.0	14.2	15.6
	Projected Change	—	2.2 °C	3.6 °C
Precipitation (PP)	Average (mm)	1,084.9	1,159.0	1,180.0
	Projected Change	—	6.8%	8.8%
Total runoff (QQ)	Average (mm)	402.5	442.7	451.9
	Projected Change	—	10.0%	12.3%
QQ/PP	Projected Change	37.1%	38.2%	38.3%
Direct runoff (DR)	Average (mm)	337.8	372.2	380.2
	Projected Change	—	10.2%	12.6%
DR/QQ	Projected Change	83.9%	84.1%	84.1%
Groundwater (GW)	Average (mm)	62.1	67.8	69.1
	Projected Change	—	9.2%	11.3%
Evapotranspiration (ET)	Average (mm)	682.4	716.5	728.0
	Projected Change	—	5.0%	6.7%
ET/PP ( = 1 − QQ/PP)	Projected Change	62.9%	61.8%	61.7%
Potential ET (PET)	Average (mm)	1,085.3	1,154.4	1,183.1
	Projected Change	—	6.4%	9.0%
Soil Moisture (SS)	Average (mm)	2,558.4	2,535.6	2,510.4
	Projected Change	—	−0.9%	−1.9%
Available Water (WA)	Average (mm)	3,634.8	3,691.2	3,687.6
	Projected Change	—	1.6%	1.5%

The values are the overall averages of projected changes of each hydrologic component (e.g. QQ) across the study watersheds.

The monthly ensemble precipitation and temperature projection made by using the 35 GCMs for the entire 61 watersheds, as well as the “03232500” watershed that was selected as an example because of its representability in terms of location (the middle of the study watershed group) and size (366 km^2^), are plotted in Figs S3 and S4, respectively. The amount of the variation in the projected precipitation did not change over time, but under RCP 8.5, the amount was more substantial than that under RCP 4.5 (Figs. S3 and S4). Under the RCP 4.5 scenario, the GCMs predicted that the overall precipitation and temperature of the Ohio Basin watersheds increased at the rates of 0.51 mm/a and 0.28 °C/a, respectively, which correspond to the slopes of the linear trend lines of Fig. S3. The rates increased to 1.25 mm/a and 0.64 °C/a for precipitation and temperature, respectively, under the RCP 8.5 scenario. As seen in Fig. S4, the variations in the precipitation ensemble are higher than those in the temperature ensemble, which indicates the projection of precipitation is more susceptible to the selection of GCMs than is that of temperature.

### Projected hydrological changes

Monthly hydrographs of the hydrological components that were created using multiple GCMs and the behavioral parameter sets of the ABCD model were averaged to construct multi-parameter and multi-GCM ensemble streamflow hydrographs for each watershed (Fig. S5; Table 1). It is worth clarifying that the ranges and heights of the boxes represent variations in the projections across the 61 study watersheds in the boxplots.

Overall, the annual averages of precipitation (PP), total runoff or streamflow (QQ), direct runoff (DR), groundwater (DR), and evapotranspiration (ET) were projected to increase when compared to those of the baseline (or historical) period under the RCP 4.5 and RCP 8.5 scenarios (Table 1). The projected increase rates of the hydrological components including QQ, DR, and GW were higher than those of PP (6.8% and 8.8% for the RCP 4.5 and RCP 8.5 scenarios, respectively), which is in agreement with projections reported in the literature^36^, indicating that the projected precipitation changes can be amplified in the runoff hydrographs (Table 1). Such amplification happens as the projected precipitation increases concentrate on winter and spring (Fig. 1(A,B)) when soil water content (SS) is relatively high (Fig. S5) in the study watersheds. The potential ET (PET) was expected to increase at rates similar to those of the TAV (average air temperature) and PP, while the ET did not change as much as did the TAV, PP, and PET due to the projected decreases of the infiltration and SS (Table 1). The available water (WA) of the watersheds was predicted to increase by 1.6% and 1.5% under the RCP 4.5 and RCP 8.5 scenarios, respectively, which implies that the overall amount of the water resources may not decrease in the future due to the projected increases in PP in the Ohio River watersheds.

Simulated ensemble hydrographs showed unique watershed wise variations depending on the hydrological component (Fig. S5). In the cases of QQ, DR, GW, PET, ET, and WA (Fig. S5), the spatial (across watersheds: the heights of boxes, and the ranges between the maximum and minimum depths) and seasonal variations in the projections made for the far future (2070 to 2079) under the RCP 8.5 scenario were larger than those in the projections provided by the RCP 4.5 scenario for the near future projection, implying higher uncertainty regarding far future hydrological projections that are under extreme emission scenarios. The projections of DR showed greater seasonal variations than those of GW, which is in agreement with our understandings, whereby the response of surface flow to rainfall is more direct than that of groundwater. The high seasonal variations found in the PET projections, ranging from 5 mm to 350 mm, were damped in the ET projections due to the interactions between soil particles and water that are expressed by the water holding capacity of soil (Fig. S5). The projections of SS and WA were widely and symmetrically distributed across the watersheds during each month, demonstrating the hydrological variety of the selected watersheds. The annual and monthly watershed hydrology projections show that the multi-GCM and multi-parameter ensemble averages could reasonably describe the overall hydrological response of the Ohio River watersheds to climate projections.

**Figure 2:**
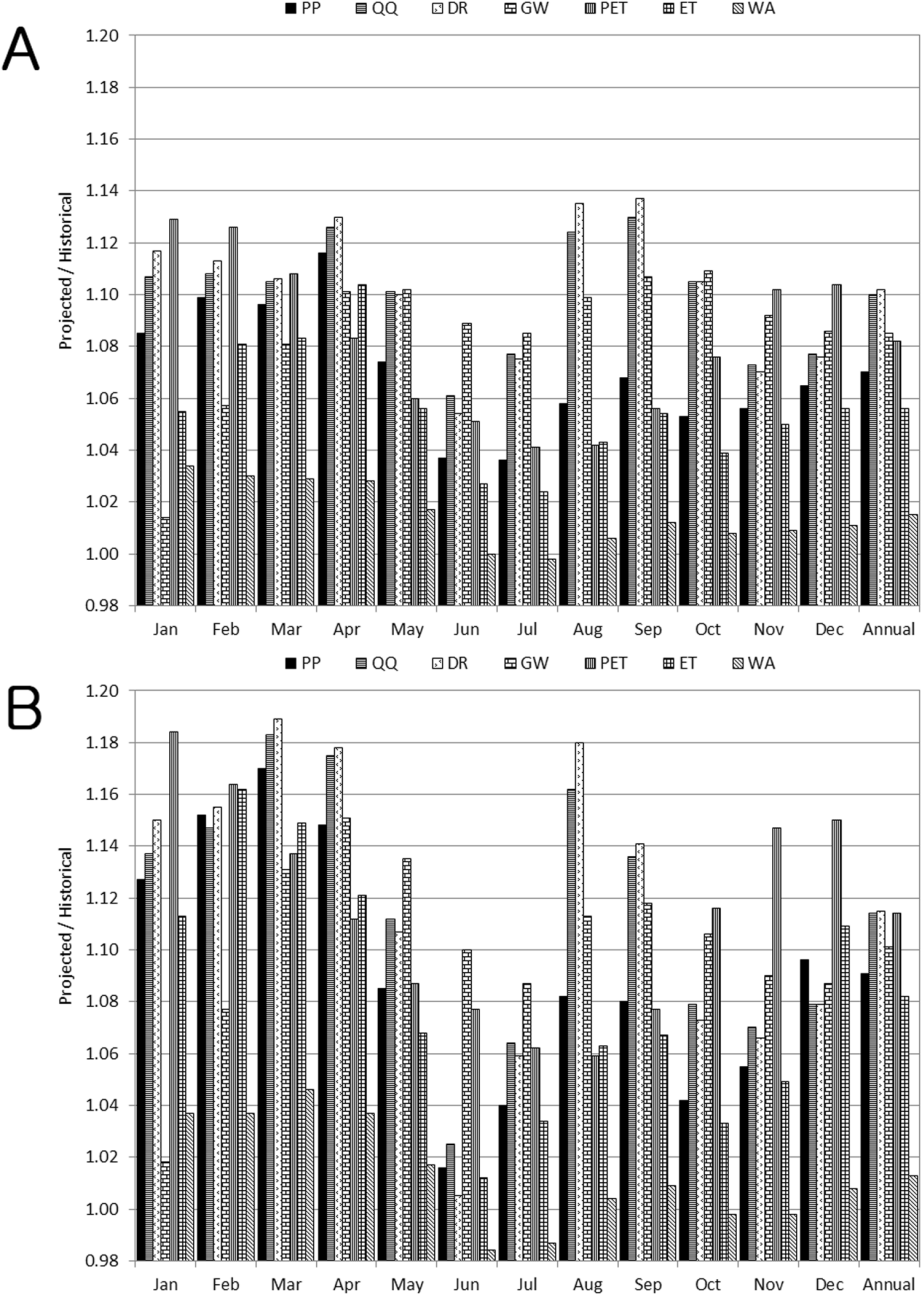
Multi-GCM, multi-parameter, and multi-watershed projections of the overall changes in the hydrologic components of the study watersheds: (**A**) RCP 4.5 and (**B**) RCP 8.5.

**Figure 3:**
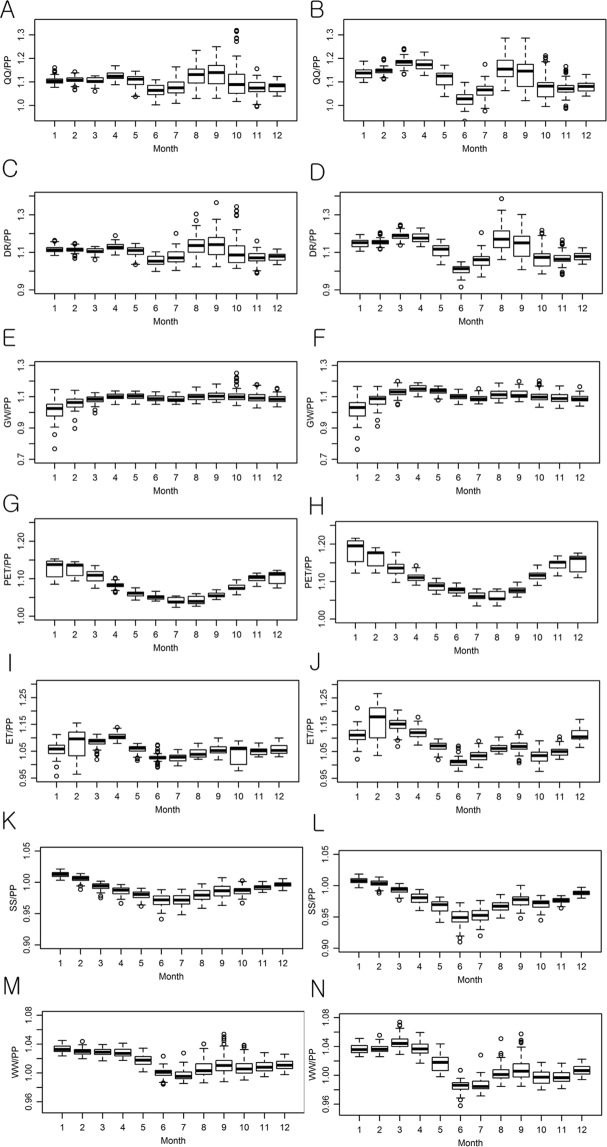
Multi-GCM and multi-parameter ensemble predictions of changes in the watersheds’ hydrologic components. Results for the RCP 4.5 and RCP 8.5 scenarios are placed in the left and right columns, respectively.

The monthly projections of the multi-parameter and multi-GCM ensembles regarding the hydrological components were compared with the historical data to attain an understanding of the overall projected seasonal changes of the Ohio River watersheds’ hydrology (Figs. 2 and 3). QQ, DR, GW, and ET were predicted to increase in all months, but SS was anticipated to decrease in most of the months, with the exception of January and February (Fig. 2). WA of the watersheds was expected to decrease in June and July under the RCP 4.5 scenario, and in June, July, October, and November under the RCP 8.5 scenario. The rates of projected QQ and DR increases were larger than those of PP for all months, indicating that the amplified climate change impact on QQ is mainly attributed to the increases of DR (Figs. 2 and 3). The projected increase of the ET was relatively large in winter and spring, which corresponds to the temperature projection. The increased ET caused a decrease in SS, with the exception of January and February in which a small amount of ET was shown, implying an agricultural drought would be deepened in the watersheds.

### Uncertainty of hydrological model parameter selection

The behavioral parameter values of the ABCD models developed for the 61 study watersheds were aggregated by the parameters to develop the overall parameter posterior distributions (Fig. S6). The mode of the posterior distribution of parameter *a* that is related to the infiltration capacity was the highest in the narrowest value range, and that of parameter *d* for the control of groundwater flow showed the lowest mode with the widest value range. Considering the hydrological meanings of *a* and *d* in the ABCD model^60^, such findings indicate the infiltration excess mechanism is dominant in the watersheds, and the proportions of groundwater to streamflow are relatively variable and uncertain across the study watersheds. The posterior distribution of *b* had a symmetric bell shape with a mode in the range from 200 to 500, meaning that the maximum monthly storage capacity of the watersheds is 350 mm on average. The parameter *c* values were distributed around 0.1, ranging from 0.0 to 0.6, indicating that the groundwater contribution to streamflow is approximately 10%, but that it is also highly variable across the watersheds. The posterior distribution of *e*, introduced to adjust the ET values, was relatively symmetric around 1.0 with a tail on the right side.

**Figure 4:**
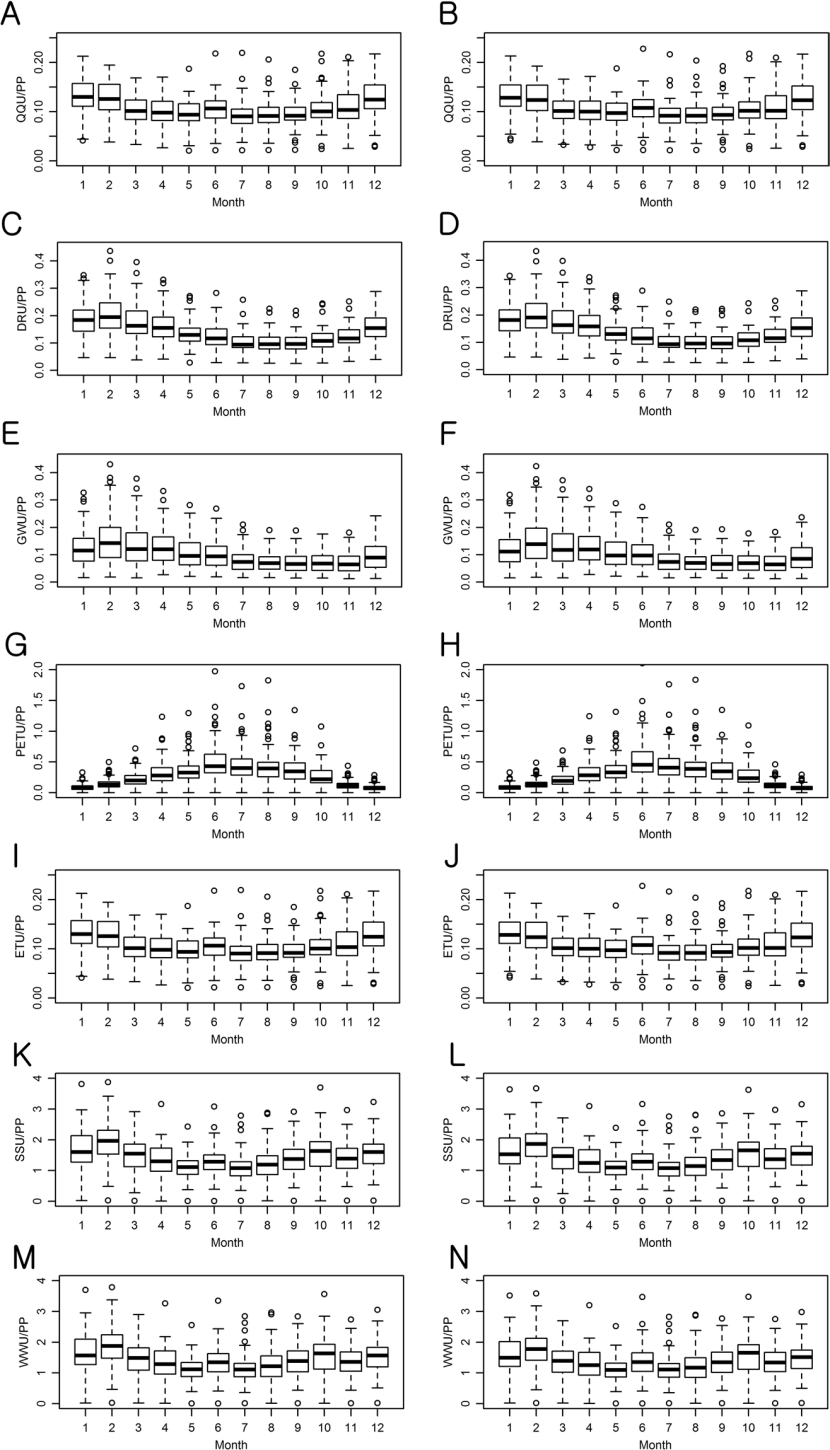
Watershed wise variations of uncertainty in the multi-parameter hydrological component ensemble projections. Results for the RCP 4.5 and RCP 8.5 scenarios are placed in the left and right columns, respectively. XXU signifies the uncertainty (U) of XX in the unit of XX (mm).

**Figure 5:**
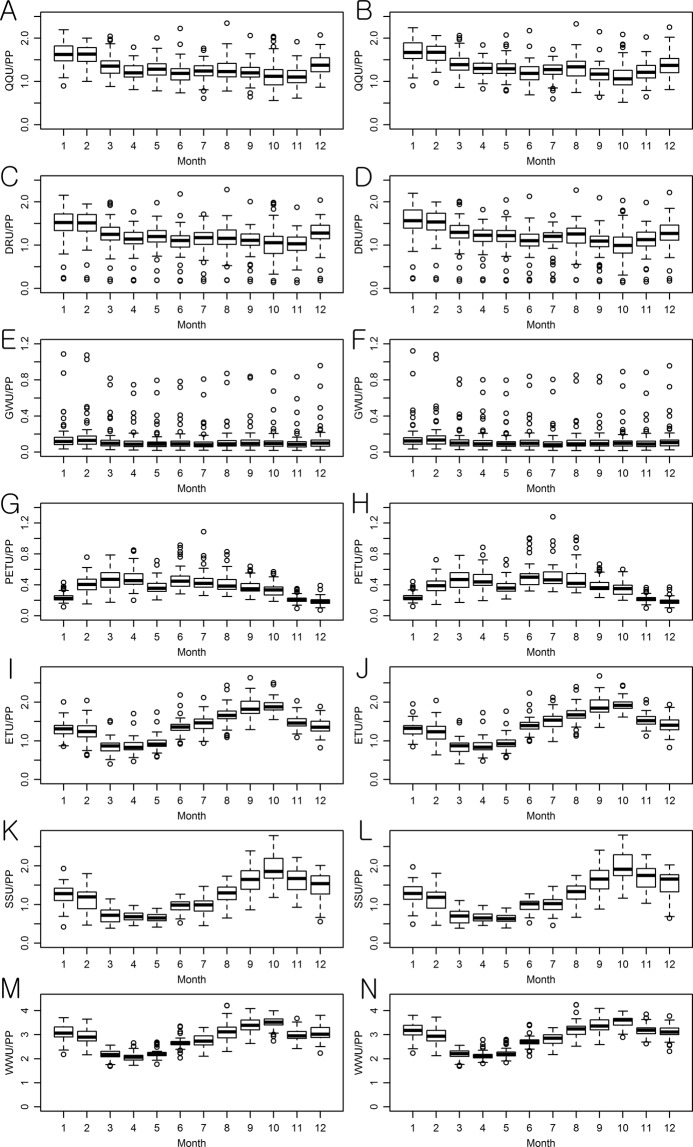
Watershed wise variations of uncertainty in the multi-GCM hydrological component ensemble projections. Results for the RCP 4.5 and RCP 8.5 scenarios are placed in the left and right columns, respectively. XXU signifies the uncertainty (U) of XX in the unit of XX (mm).

Uncertainty in the projections of the multi-GCM and multi-parameter ensembles of hydrological components was first quantified in the depth unit of mm by the study watersheds; then, it was normalized by dividing their depths by the precipitation depths for the purpose of a fair comparison of uncertainty across the watersheds (Figs 4 and 5). It is worth clarifying that the average values represent the overall uncertainty in the Ohio River watersheds and the height of each box represents the watershed wise variations of the uncertainty in the boxplots (Fig. 4).The overall average uncertainty in the monthly multi-parameter ensemble streamflow (QQ) projections for the 61 study watersheds varied from 9.2% (8.63 mm) to 13.4% (11.93 mm) of monthly precipitation depths under RCP 4.5 (Fig. 4). Variations of the streamflow projection uncertainty across the watersheds were relatively large in winter; no significant difference was found in the amount of uncertainty between the QQ projections under RCP 4.5 and RCP 8.5 scenarios. The amount of uncertainty in the QQ projections was smaller than those of the DR projections, but they were larger than those in the GW projections, indicating that DR is more sensitive to parameter uncertainty than GW.

The PET projection showed a more considerable uncertainty compared to those of QQ, DR, or GW, particularly in summer. The PET projections also showed great spatial variations across the latitudes between 36° 07′N and 42° 26′N within the Ohio River basin; alternatively, the uncertainty of the actual ET projections was larger in winter than in summer, but the seasonal variations were small. Low SS could limit ET in summer, which regulated the variations of the uncertainty in the ET projections. Moreover, compared with summer, the ET projection variations were more substantial during winter when SS was relatively high. Uncertainty in the SS projection did not largely vary by the seasons due to the water-holding capacity of the soil layers. Since WA mainly consisted of SS, the amounts and seasonal trends of the uncertainty amounts of each were similar.

### Uncertainty in climate model selection

**Figure 6:**
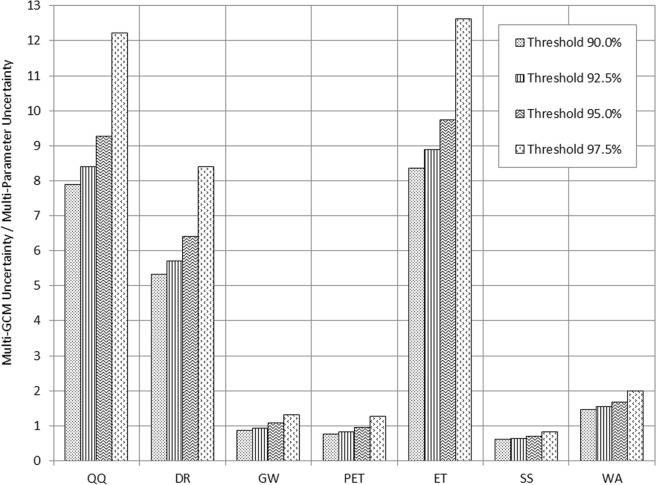
Sensitivity of uncertainty quantified for multi-parameter hydrologic component ensembles to behavioral parameter thresholds.

**Figure 7:**
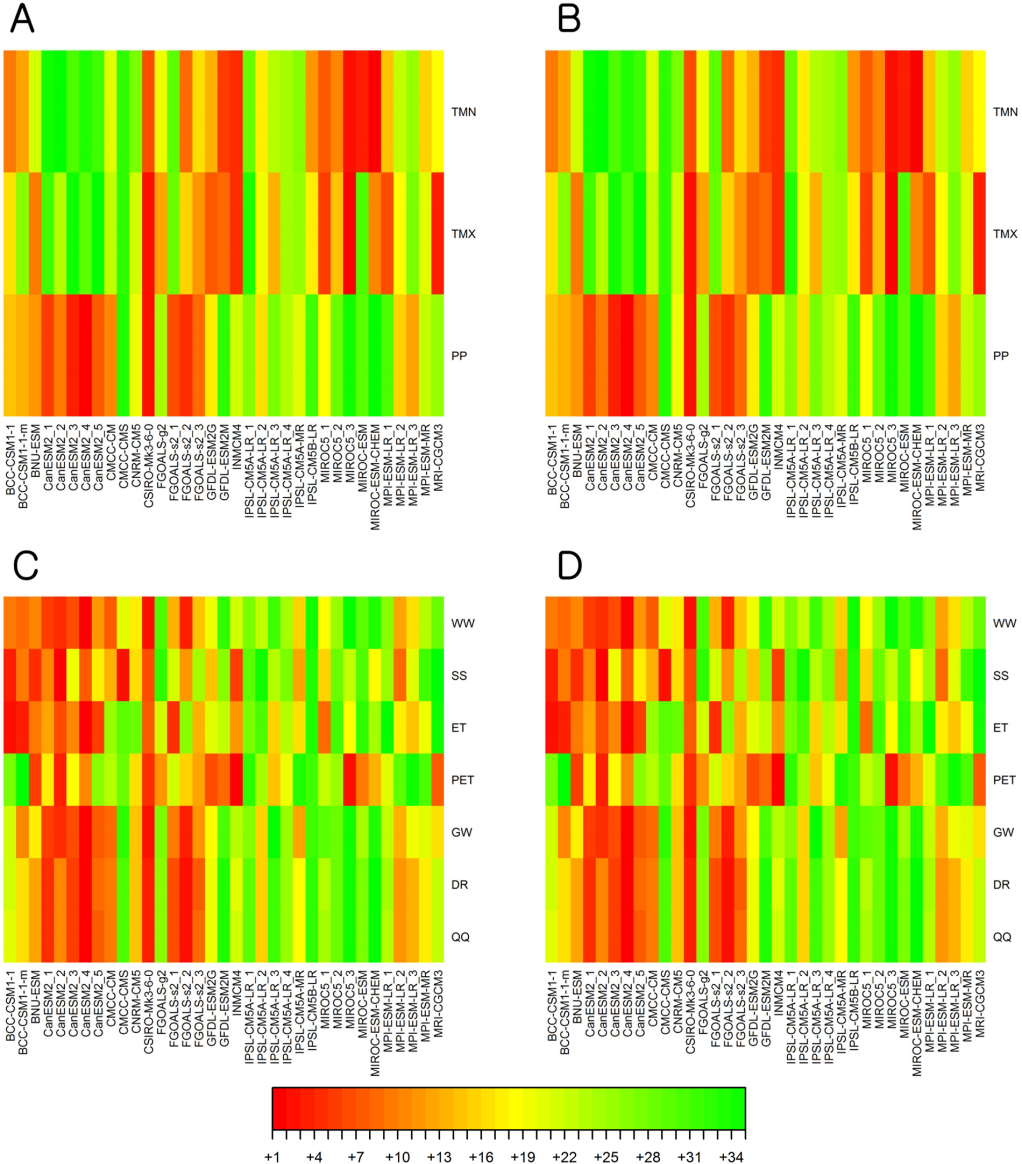
GCM contributions to uncertainty in the multi-GCM ensemble projections made for climate variables and hydrologic components. The numbers represent the ranks of GCMs’ contributions to the overall uncertainty (from the largest contribution to the smallest). Results for the RCP 4.5 and RCP 8.5 scenarios are placed in the left and right columns, respectively. Under RCP 4.5, for instance, IPSL-CM5B-LR provide the least amount of uncertainty in the multi-GCM ensembles for all the hydrologic components, and CSIRO-MK3-6-0 gave the largest as it created the most significant uncertainty in the multi-GCM ensembles of the climate variables including PP.

The selection of climate model was an order of magnitude more influential on uncertainty in the QQ, DR, and ET projections than that of parameter selection, but it was not always the case for GW and PET (Figs. 4 to 6). In the case of QQ, the overall average uncertainty in the monthly multi-GCM ensemble projection ranged from 113% (99.3 mm) to 164% (160.4 mm) of monthly precipitation under RCP 4.5 (Fig. 5). Uncertainty in the QQ projections was greater in winter than in other seasons, and it was dominated by the GCM selection uncertainty in the DR projections. The GW prediction was relatively less responsive to the GCM selection compared to those of QQ, DR, and ET (Figs. 5 and 7). The GCM selection was much more influential on the ET projection than the PET since ET is controlled by not only temperature but also SS that is sensitive to GCM selection. The monthly variation patterns of uncertainty in the PET projections were opposite to those of the DR projections in both the multi-GCM and multi-parameter ensemble cases, implying that the impact of the PET prediction uncertainty on DR projections is limited (Figs. 4 and 5). Uncertainty in the WA projections due to the GCM selection was 2 to 4 times larger than uncertainty in the PP projections, indicating the significance of GCM selection in a climate change impact analysis.

As the threshold values for the identification of behavioral parameter sets increased from 90.0% (a relatively conservative threshold for equifinality quantification) to 97.5% (a relatively liberal threshold), uncertainty in the GCM ensembles and its relative size to the uncertainty in the parameter ensembles increased exponentially (Fig. 6). In the case of SS, parameter selection was more critical than GCM selection in all of the threshold cases. When relatively loose thresholds (i.e. 90.0% and 92.5%) were used, the selection of the hydrological model parameters became more significant than the GCM selection for the GW and PET projections, implying that the selection of hydrological model parameters needs to be more careful than that of GCMs when soil moisture and groundwater are the concerns of a climate change impact study. Since ET is determined as a direct function of precipitation and direct runoff in the ABCD model, the corresponding uncertainty in the ET projection would become as significant as the uncertainty in the QQ projections (Fig. 6). Depending on the thresholds, the GCM selection was 1.5 to 2 times more influential than the parameter selection with respect to an assessment of the climate change impacts on the amount of available water in the watersheds.

The contribution of each GCM to the uncertainty in the GCM ensembles varied depending on the types of hydrological components (Fig. 7). Overall, the amount of uncertainty contributed by the climate change projections made using BCC, GCESS, CCCMA, CSIRO-QCCCE, and LASG-CESS for the Ohio River Basin watersheds were larger than those of INM, IPSL, and MIROC. Furthermore, uncertainty in the precipitation projections made by some GCMs was large while uncertainty in their temperature projections was small, and vice versa. It will be useful to identify which models contribute the most to the uncertainty of an ensemble projection, as we can focus on such models first when trying to reduce uncertainty contained in the ensemble projection by screening unrealistic modeling results. In addition, when it is not feasible to include all GCMs and their projections in a climate change impact study and its uncertainty analysis, the uncertainty information such as Fig. 7 can provide guidance to researchers to make appropriate and fair model selections. On the other hand, uncertainty in the following three GCMs was relatively small for both climate variables in the Ohio River watersheds: CMCC-CMS, IPSL-CM5A-LR_1, and IPSL-CM5A-LR_4.

**Figure 8:**
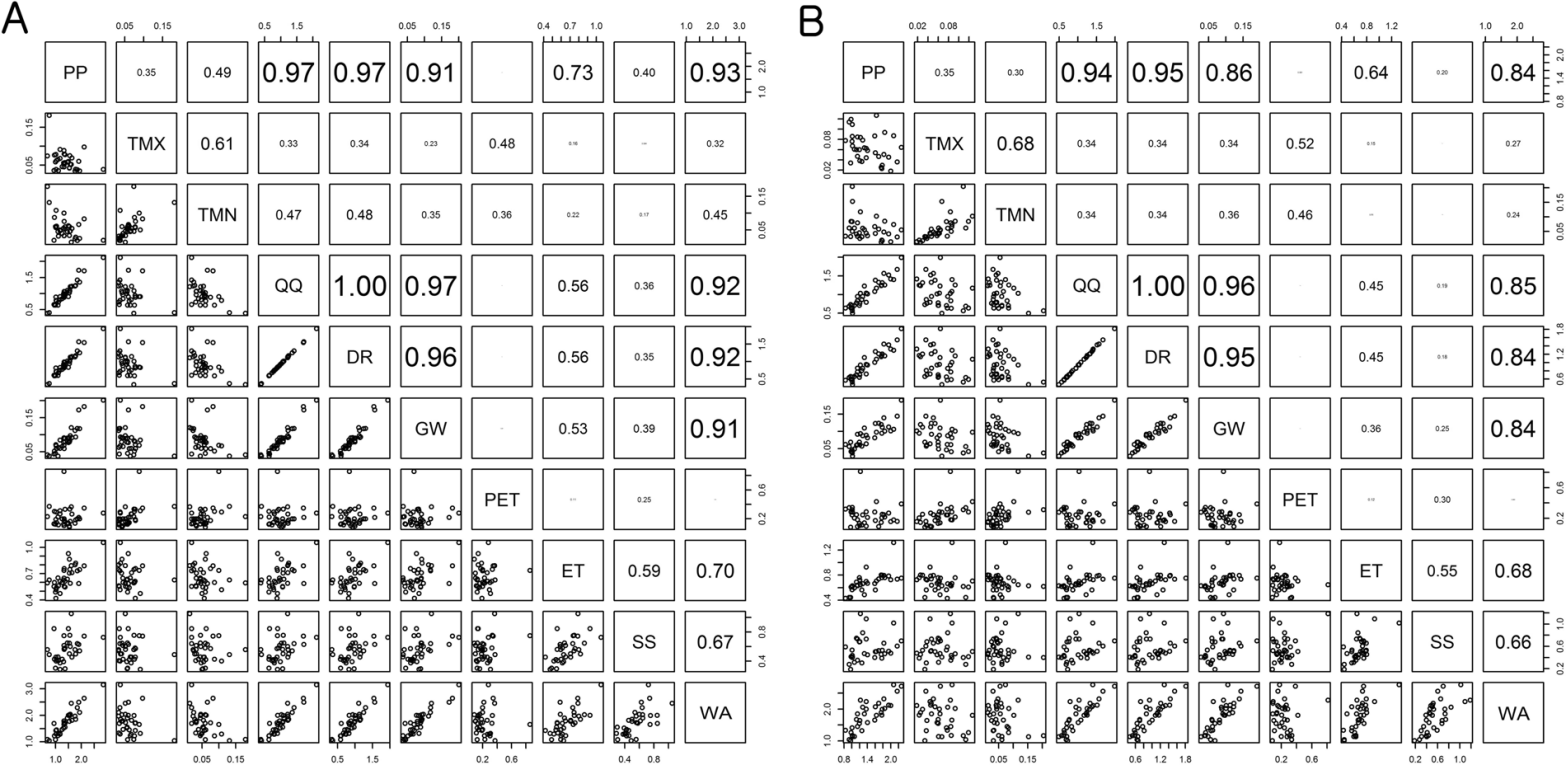
Correlation between the amount of uncertainty in the projections for climate variables and hydrologic components: (**A**) RCP 4.5 and (**B**) RCP 8.5.

The amount of uncertainty in the GCM ensemble projections for the hydrological components and precipitation were highly correlated to each other (Fig. 8). For example, the amount of uncertainty in the precipitation ensemble was related to those of the QQ, DR, GW, and WA projections with correlation coefficients greater than 0.75. Such a finding indicates that uncertainty in the precipitation ensemble was transferred to the hydrological simulation and suggests that a more considerable effort needs to be invested in improving the projection accuracy of precipitation than temperature in a hydrological analysis of climate change (Fig. 8). PET was moderately correlated with maximum air temperature (TMX), minimum air temperature (TMN), and TAV, and this reflects the characteristics of the Hargreaves equation that was used for the PET calculation of this study.

**Figure 9:**
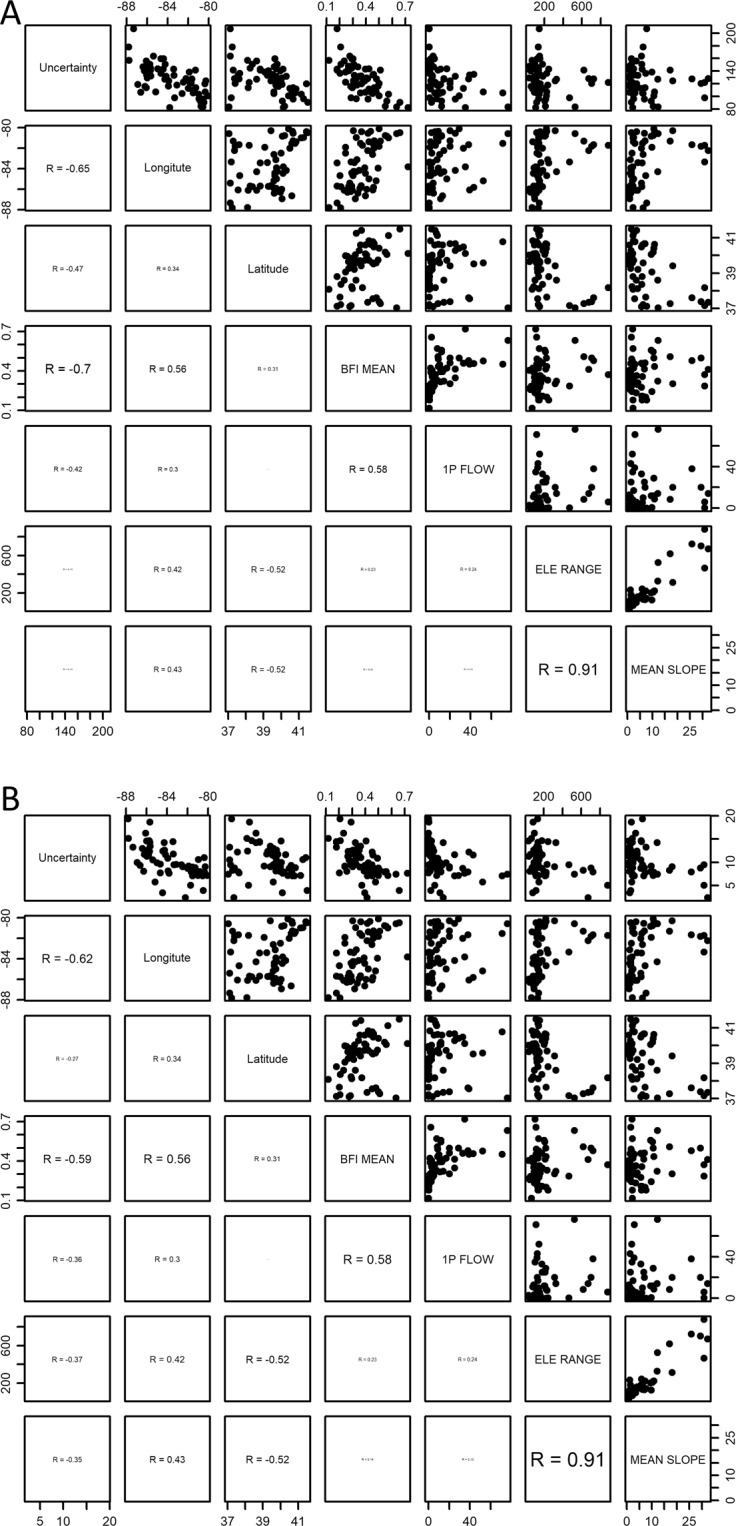
Correlation between the amount of uncertainty in QQ (total runoff or streamflow) projected under RCP 4.5 and the selected hydrological characteristics of the watersheds: (**A**) Multi-parameter uncertainty and (**B**) multi-GCM uncertainty. “Uncertainty”: the amount of the multi-parameter or multi-GCM uncertainty, “BFI MEAN”: average annual baseflow index (BFI) values, which represent the fraction of baseflow in streamflow (or total runoff), “1 P Flow”: the 1^st^ percentile daily streamflow value, “ELE RANGE”: the difference between elevations of the highest and lowest points within a watershed, “MEAN SLOPE”: the average slope (%) of a watershed.

The correlation structure between the amount of uncertainty in QQ and the selected hydrological features of the watersheds was explored to see if uncertainty magnitudes can be predicted on the basis of watershed characteristics (Fig. 9). Both multi-parameter and multi-GCM uncertainty were moderately (0.5 < R < 0.8^61^) correlated to the longitudes of the mass centers of the watersheds and the fraction of baseflow in streamflow. As seen in Figs. S1 and S2, there are gradual changes in climate and the degree of projected temperature increases from southwest to northeast. The correlation analysis shows that uncertainty magnitude decreases as moving to the east of the basin (longitudes increase from −88° 30′ to −78° 00′), indicating a mild temperate or humid subtropical climate (southwest) may have relatively large uncertainty in hydrological analysis of climate change than a humid continental climate (northeast) in the basin. In addition, uncertainty was relatively small in watersheds with high average annual baseflow indices (BFIs), which well corresponds to Figs. 4 and 5. In the figures, the amount of multi-parameter and multi-GCM uncertainty associated with groundwater (GW) is relatively small compared to those of the other hydrological components.

## Discussion

This study systematically compared the influence of uncertainty in temperature and precipitation projections on various hydrological variables at the local watershed scale. The results showed that the uncertainty of GCM projections are dominant for relatively rapid hydrological components while the uncertainty of parameterization is more significant for slow components. The study demonstrated that all GCMs (rather than a few one) contribute uncertainty in multi-GCM ensemble predictions, and their contributions vary by watersheds and months, suggesting the needs for the reliability assessment of GCM projections when developing watershed-scale management plans. The findings also suggest that the selection of both GCMs and parameters should be carefully made to improve the robustness of a hydrological assessment of climate change.

**Figure 10:**
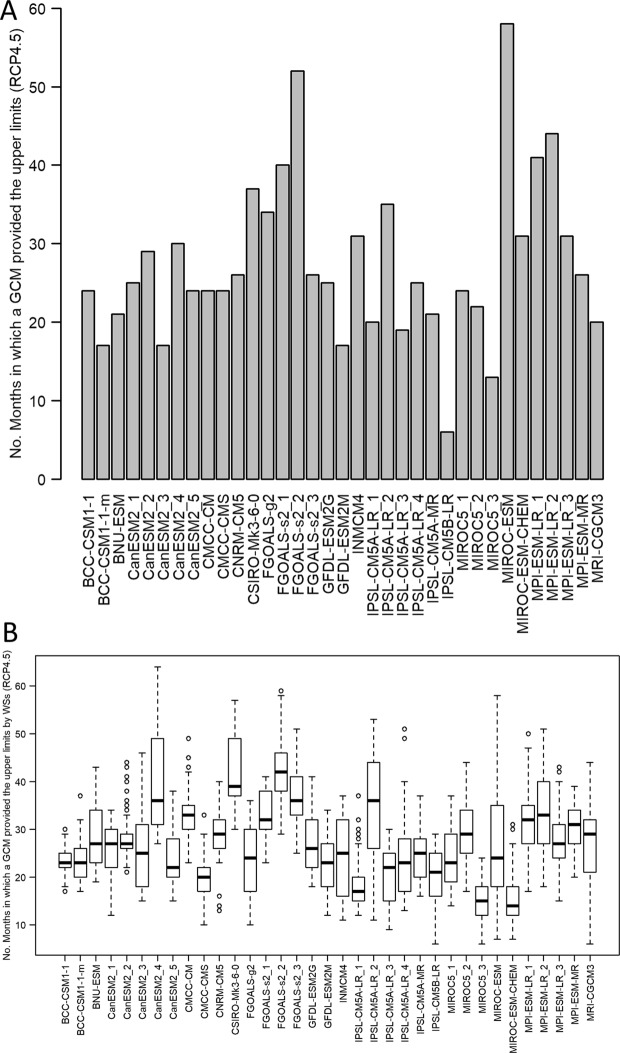
Varying contributions of GCMs to multi-GCM ensemble predictions. (**A**) The numbers of months in which each GCM provided the upper/lower limits of the WA (available water) projections for “03232500” (the summation of the numbers is equal to 960 months). (**B**) Variations in the numbers of months in which each GCM provided the upper/lower limits of the WA (available water) projections made for the watersheds (the number of data points in each box-whisker column is equal to 61 watersheds).

GCMs that contribute to the uncertainty of hydrological projections vary by months (as Equation 9 is applied to individual months). In the case of “03232500”, for instance, the numbers of months in which each GCM provided the upper limits vary as shown in Fig. 10A. In addition, Fig. 10B shows variations in the numbers of months in which each GCM provided the upper limits of the WA projections made for the watersheds. Such results demonstrate that all GCMs (rather than a few ones) are partially responsible for uncertainty in multi-GCM ensemble predictions, and their contributions to uncertainty vary by months.

The uncertainty of hydrological projections, especially for runoff, was much more closely correlated to the uncertainty of projections for precipitation than temperature, suggesting that the reliability of the precipitation projections made by GCMs needs to be investigated for a robust hydrological analysis of climate change. Overall, the results demonstrate how a subjective selection of climate models and hydrological model parameter values can affect the hydrological assessment of climate change and highlight the importance of quantitative uncertainty analysis for improved reliability of the assessment. We also could examine the contributions of each GCM to uncertainty in a multi-GCM ensemble. The GCM uncertainty contributions quantified using the newly introduced analysis strategy would be useful information and indicator to screen GCMs in creating precise ensemble hydrological projections. The selection of GCMs can be guided by various information including the amount of uncertainty in projections, the accuracy of reproducing historical data (or observations), perceived accuracy of climate models (based on an understanding of the simulation mechanisms), and the overall performance reported in literature. This study presents a method to quantify and compare the contribution of GCMs to uncertainty in precipitation and temperature projections, as demonstrated in Fig. 7A. The information provided in the figure is expected to help to screen GCMs that increase uncertainty in hydrological analysis of climate change.

This study showed that relatively fast hydrologic components such as direct runoff are more sensitive to the uncertainty of the GCM ensemble compared to the equifinality of the hydrological model. Thus, the impact of uncertainty in ensemble precipitation and temperature projections on the hydrological analysis of climate change are expected to be larger in a small local watershed whose hydrological responses tend to be dominated by direct runoff. Some GCMs produced greater uncertainty in the hydrological projections than other GCMs, but an investigation on the relationship between the characteristics of climate models and their contributions to the overall uncertainty was beyond of the scope of this study. We employed a statistical bias-correction method developed by Ho *et al*.^62^ in this study; thus the use of different bias-correction methods may lead to different analysis results. However, we believe it won’t change our conclusion as the differences between data bias-corrected by statistical and dynamical downscaling methods are not substantial when the same GCMs were employed, especially at a monthly scale^63–65^.

This study included watersheds whose hydrological responses to rainfall and temperature could be reproduced and explained with a simple water balance model, ABCD. Thus, the results may not apply to watersheds to which the model is not applicable. Although the applicability of the ABCD model to the Ohio basin has been proven^66^, and only ABCD models successfully calibrated to streamflow were included in this study, the representation of water balance partitioning varies by models. Thus, a hydrological model different from ABCD will provide simulation results different from what this study obtained. It may be worth investigating whether the use of other hydrological models calibrated at an accuracy level similar to that of this study may affect the conclusions (model structure equifinality vs. model parameter equifinality). However, such examination is beyond the scope of this study.

ABCD is a spatially lumped, continuous monthly hydrological model. In the model, the water balance partitioning of a watershed is defined by nonlinear mathematical relationships between hydrological components. The model representation of the partitioning was calibrated to streamflow measurements made at the watershed outlets in this study. Out of 156 watersheds that have streamflow measurements in the Ohio basin, in addition, only 61 watersheds where the ABCD model provided the minimum model performance statistics of NSE of 0.67 were included in the analysis. The ABCD model has been shown to be capable of re-enacting covariance structure found in between observed PP, PET, and QQ in validating the applicability of the model to watersheds in the U.S.^67^. The ABCD model provided good performance in describing the water balance partitioning of the energy-limited (rather than water-limited) areas including the Ohio basin^66^. Recently, a study has found that the ABCD model could describe the monthly water balance of an extremely arid watershed and its annual variations^68^. Baseflow fractions simulated using the calibrated ABCD models were compared with the observed to confirm its accuracy of reproducing the water balance partitioning (Fig. 5). The threshold NSE of 0.67 was chosen for the watershed selection based on literature (Moriasi *et al*.^69^; Engel *et al*.^70^; Martinez and Gupta^66^; Ritter and Munoz-Carpena^71^). In the studies, the NSE value of 0.50, 0.65, or 0.67 was reported or suggested as a lower limit of valid goodness-of-fits commonly used in hydrological modeling. We selected the NSE value of 0.67 close to the lower limits (for ‘acceptable’ performance rating) reported in the studies.

Although the literature review, parameter calibration, and watershed screening ensured the applicability of the model to the study watersheds, still there must be uncertainty and errors in the modelling results due to the conceptual nature of the model, the lack of observations showing detailed hydrological partitioning processes, and the limitations of the calibration and uncertainty analysis methods. For instance, the lumped representation of the watershed processes may not effectively regulate parameter values in the calibration. The use of a spatially distributed and/or process-based approach can improve the identifiability of calibration parameters and then reduce output uncertainty caused by parameter equifinality^41,72–74^. Even when a hydrological model that employs more spatially explicit and sophisticated simulation mechanism is used, observations that can identify the partitioning of internal hydrological components such as direct runoff, soil water content, and groundwater recharge (rather than streamflow) would be necessary to constrain calibration parameters. The additional model complexity that is not supported by such ground truth can further increase output uncertainty. Studies have demonstrated that there is no clear and simple relationship between model complexity and output uncertainty, which must be attributed to the unique structures of models used, the availability of observations for parameter calibration, and the landscape characteristics of a study area of interest^41,74,75^.

The sampling-based optimization algorithm, Shuffled Complex Evolution – University Arizona (SCE-UA), was used to identify parameter sets providing acceptable performance in this study, and the algorithm has been known to be capable of locating a solution close to the global optimum^76–80^. The heuristic features of the algorithm could reveal the equifinality of the model’s watershed representation, but the ranges of identified parameter values would be dependent on calibration algorithms used due to their unique sampling strategies and subsequent efficiency^41,81,82^. In addition, there has been a debate about the efficiency, statistical formality, objectivity, and soundness of uncertainty analysis frameworks commonly used^48,83,84^. Studies proposed strategies, approaches, models, and methods to solve them, and still many of them remain as subjects for next studies^83,85^.

The use of different models, calibration and uncertainty analysis methods may bring results different from what we have here. Assuming that observations are only enough to regulate major parameters in calibration, however, overparameterization is likely to increase equifinality and uncertainty^42–44,86,87^. The parsimonious structure of a simple model such as ABCD can minimize the possibility of overparameterization and then reduce parameter uncertainty. Thus, the amount of multi-parameter uncertainty quantified in this study may represent one of the optimistic cases on the side of hydrological modeling. Such a speculation implies that the use of more process-based models that usually have a relatively large number of parameters may increase hydrological modeling uncertainty especially when additional measurements that can constrain model behavior are not incorporated in parameter calibration.

In this study, the behavioral parameter sets were selected by comparing runoff observed and simulated using a rainfall-runoff model (i.e. ABCD), which is part of hydrological model parameter calibration. Then, the calibrated or behavioral models are considered capable of representing the runoff generation mechanisms of study watersheds, which may not change easily even for a long time. In the study, changes in land uses (e.g. urbanization and deforestation) and topography (by erosion and sediment transport) are assumed not to be significantly large enough to alter the runoff generation mechanism in the study period. Changes in climate may alter the mechanism, but they are also assumed insignificant so that the calibrated parameter values and behavioral models can be valid for hydrological analysis of future climate change in this study.

A total of 22 GCMs and their variants were considered in this study so that the wide ranges of mathematical representations and climate process simulation strategies could be considered, and the largest uncertainty in the multi-GCM ensembles could be explored. When the amount of uncertainty is expressed in the depth unit (mm) at monthly and annual scales, it was turned out that uncertainty in hydrological component projections made using the multi-GCM is considerably large compared to the rainfall depth projected, indicating a GCM selection can substantially affect the hydrological analysis of climate change (Fig. 5). Such finding suggested that a map showing the ranges (uncertainty) and trends of the precipitation and temperature projections should be built using multiple GCMs, or hopefully all of them—which are used in the global scale climate projections for watersheds (e.g. 8- or 12-digit Hydrologic Unit Code watersheds)—to guide the field of hydrological modeling for more effective GCM selection in the climate change studies for local watersheds.

## Methods

### Study area

**Figure 11:**
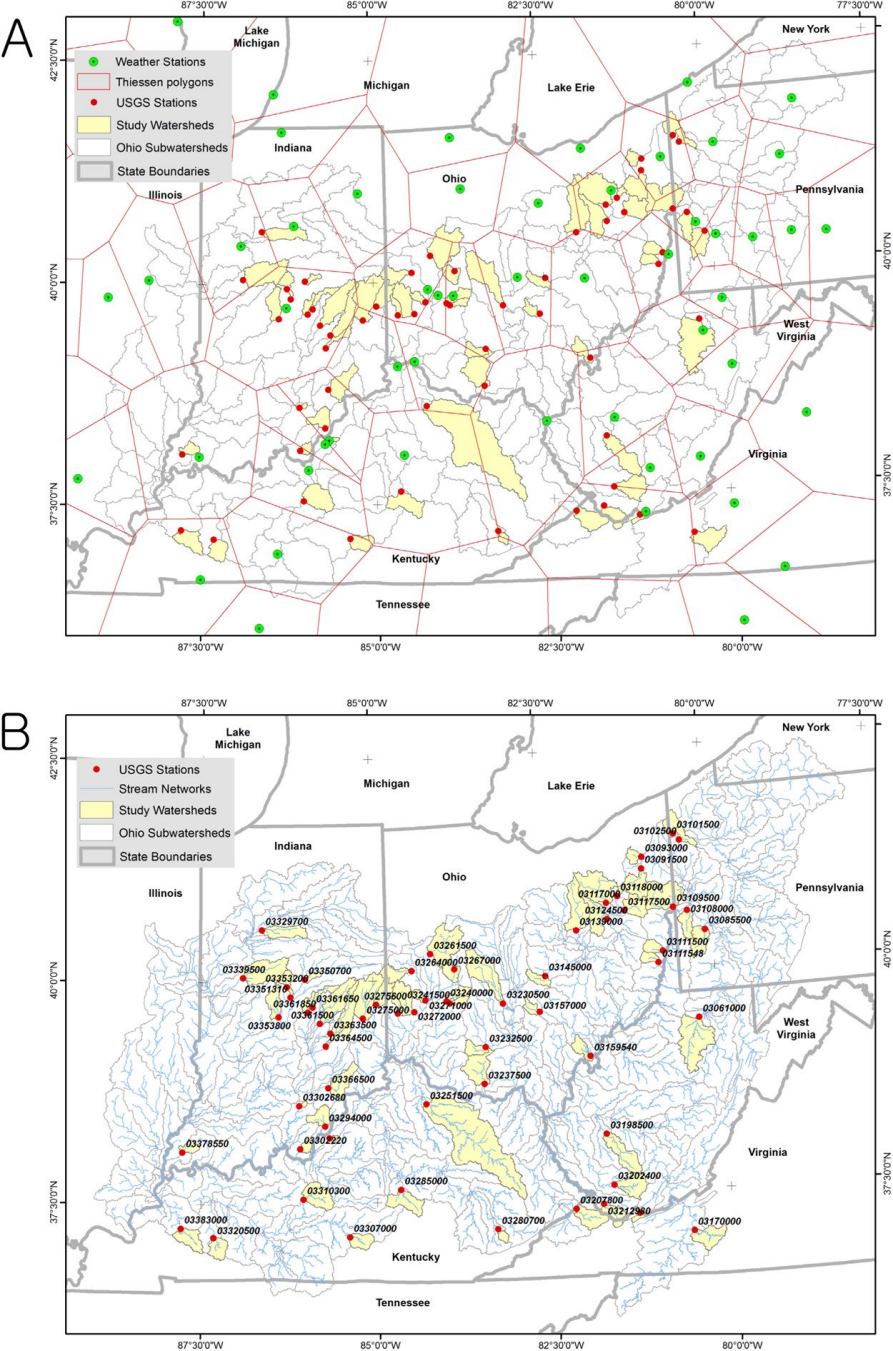
Study watersheds in the Ohio River basin: (**A**) the weather stations into which GCM data were bias-corrected and their Thiessen polygons, (**B**) the stream networks and USGS gage stations at which the ABCD model parameters were calibrated.

The Ohio River basin is located in the Eastern Corn Belt and extends across nine states from Illinois to New York, between the latitudes 36° 07′ and 42° 26′ North, and the longitudes 77° 50′ and 89° 01′ West (Fig. 11). Climate varies from a mild temperate or humid subtropical (southwest) to humid continental (northeast) within the basin. The basin drains a primarily agricultural area of 374,000 km^2^ including several large cities into the Mississippi River and eventually the Gulf of Mexico. For this study, 61 study watersheds within the Ohio River basin were selected for the consideration of the drainage areas, geographic locations, climate, availability of streamflow measurements, and applicability of the water balance model, ABCD (Fig. 11, also refers to a multi-parameter ensemble). The total drainage area of the selected watersheds is 41,341 km^2^, (average size is 678 km^2^), which are equivalent to 11% of the entire Ohio River basin. Daily precipitation and temperature records made at 103 weather stations associated with the basin were used to correct biases in GCM climate projections.

It has been predicted that the annual streamflow of agricultural watersheds located in the Midwestern U.S. could decrease by as much as 40% depending on GCMs under the Special Report on Emission Scenarios (SRES)^88^. A study found that the crop productivity of the Ohio River basin could decrease by 20% under climate projections; it also showed that there was a large amount of uncertainty in sediment and nutrient loads projected by seven CMIP5 GCMs^89^. The impacts of future climate changes on the hydrology of the Upper Scioto River Basin that drains 8,337 km^2^ into the Ohio Basin have been investigated; depending on GCMs used, the annual streamflow projections varied by a factor of two to three, which indicates a considerable amount of uncertainty in the multi-GCM ensembles made for the study basin^90^. In addition, wide variations were found in annual precipitation and temperature projections made for the Midwestern U.S.^58^.

### Multi-GCM ensemble

Over the last several years, climate projections from GCMs participated in the CMIP3 and CMIP5 have been employed for climate change impact assessments at both regional and local scales^91^. CMIP5, the latest climate data, is expected to promote multi-GCM frameworks by providing a range of projected climate sciences^4^. In this study, climate projections made for the weather gage stations associated with the study watersheds were obtained by bias-correcting the 35 climate model outputs selected from 22 GCMs of the CMIP5 (Fig. 11 and Table 2). Two RCP scenarios, RCP 4.5 and RCP 8.5, that have been commonly adopted as forcing scenarios for the CMIP5 GCMs were employed to consider uncertainty in the future social conditions^92^, leading to the formulation of 70 climate projections (35 climate models and 2 RCP scenarios) for each of the watersheds in this study (Table 2).

**Table 2:** CMIP5 GCM models and their variants used in this study (http://cmip-pcmdi.llnl.gov/cmip5/availability.html).

Model name	Realization number*	ID number	Institute ID	Resolution	Country
BCC-CSM1.1^a^	1	1	BCC	64 × 128	China
BCC-CSM1.1-m^a^	1	2		160 × 320	
BNU-ESM^a^	1	3	GCESS	64 × 128	China
CanESM2^a^	1,2,3,4,5	4–8	CCCMA	64 × 128	Canada
CMCC-CMS^b^	1	9	CMCC	96 × 192	Italy
CMCC-CM^b^	1	10		240 × 480	Italy
CNRM-CM5^b^	1	11	CNRM-CERFACS	128 × 256	France
CSIRO-Mk3.6.0^a^	1	12	CSIRO-QCCCE	96 × 192	Australia
FGOALS-g2^a^	1	13	LASG-IAP	108 × 128	China
FGOALS-s2^a^	1,2,3	14–16	LASG-CESS		
GFDL-ESM2G^a^	1	17	NOAA GFDL	90 × 144	USA
GFDL-ESM2M^a^	1	18			
INM-CM4^a^	1	19	INM	120 × 180	Russia
IPSL-CM5A-LR^a^	1,2,3,4	20–23	IPSL	96 × 96	France
IPSL-CM5A-MR^a^	1	24		143 × 144	
IPSL-CM5B-LR^a^	1	25		96 × 96	
MIROC5^a^	1,2,3	26–28	MIROC	128 × 256	Japan
MIROC-ESM^b^	1	29		64 × 128	
MIROC-ESM-CHEM^b^	1	30			
MPI-ESM-LR^b^	1,2,3	31–33	MPI-M	96 × 192	Germany
MPI-ESM-MR^b^	1	34			
MRI-CGCM3^b^	1	35	MRI	160 × 320	Japan

^a^calendar: 365 days (without a leap day).

^b^calendar: Standard (with a leap day).

*“realization” number is used to distinguish among the members of an ensemble typically generated by initializing a set of runs with different, but equally realistic, initial conditions.

As systematic biases are inevitably introduced in climate modeling, GCM outputs are often bias-corrected using observational information such as station-based and gridded observation data. In addition, because of the inconsistency regarding the spatial resolutions between GCM data and a climate change impact assessment, GCM data are often downscaled to finer resolutions, and often into existing weather stations^93,94^. For this study, the CMIP5 GCM outputs (precipitation, maximum and minimum temperature) of the Ohio River study watersheds were statistically bias-corrected over the period from 1950 to 2099 using a hybrid semi-parametric approach proposed by Ho *et al*.^62^. This bias-correction method is considered computationally efficient and easy to implement^95^. Using Eq. 1, the method matches the location (mean), scale (variance), and shape (skewness) parameters of GCM outputs with those of the historical observations to preserve the consistency of their statistical features over long-term periods:

1 $${\hat{X}^{\prime} }_{m}={\mu }_{o}+\frac{{\sigma }_{o}}{{\sigma }_{m}}({X^{\prime} }_{m}-{\mu }_{m})$$

where X, μ, and σ respectively represent the variable of interest, mean, and standard deviation of a climate, the subscripts of o and m respectively signify the observable and simulated climate variables of interest, the superscript of “**′**” indicates a future time period, and the symbol of “ ^” represents a bias-corrected variable. The bias correction approach used in this study assumes that discrepancies between observed and modeled climate variables do not change over time or in the future^62,95^. Thus, future observables could be directly predicted based on historical observations using a transfer function that maps simulated climate onto observations. The transfer function was estimated by matching the predicted future probability distributions of climate variables to their empirical (historical) distributions (Eq. 1). Then, the multi-GCM ensemble averages of precipitation, temperature, and hydrological components such as direct runoff and groundwater were determined by averaging the bias-corrected projections with equal weights, which is often called the “one model, one vote” weighting scheme.

### Hydrologic model

A monthly water balance model, ABCD was prepared to simulate the long-term hydrological responses of the 61 study watersheds to projected climate changes^60^. The ABCD model has a parsimonious structure requiring only five parameters and allowing computationally affordable long-term simulation of hydrological components of interest. The modeling capacity of ABCD satisfied our needs for the monthly simulation of hydrological components including direct runoff, soil water, evapotranspiration, and groundwater and their water balance partitioning. The model has been successful in hydrological analyses for various climate zones^37,66,96–100^. In the ABCD model, available water (*WW*, mm) of the current month is defined as a summation of precipitation (*PP*, mm) of the current month (*t*) and soil water content (*SS*, mm) of the previous month (*t* − 1) (Eq. 2), while the evapotranspiration opportunity of the current month (*YY*, mm) is determined by a summation of actual evapotranspiration and soil water content of the current month (Eq. 3):

2 $$W{W}_{t}=P{P}_{t}+S{S}_{t-1}$$

and

3 $$Y{Y}_{t}=PE{T}_{t}+S{S}_{t}=\frac{W{W}_{t}+b}{2a}-\sqrt{{(\frac{W{W}_{t}+b}{2a})}^{2}-\frac{W{W}_{t}b}{2a}}$$

where the *a* and *b* parameters represent “propensity for runoff to occur well before the soil is saturated to capacity” (0 ≤ *a* ≤ 1) and “upper limit of storage in the unsaturated zone above the groundwater level,” or “upper bound of the summation of actual evapotranspiration and soil moisture storage,” respectively^60^. *PET* represents the potential evapotranspiration (mm) that is calculated using Eq. 4:

4 $$PET=e\cdot PE{T}_{EQ},$$

where *e* is a calibration parameter that is newly introduced to the original ABCD model, and *PET*_*EQ*_ is the potential evapotranspiration estimation provided by the *PET* equation. The Hargreaves equation was selected for the calculation of the monthly *PET* in this study (Eq. 5):

5 $$PE{T}_{EQ}=0.000938(TAV+17.8){(TMX-TMN)}^{0.5}{R}_{a},$$

where *TAV* is average monthly temperature (°C), *TMX* is maximum monthly temperature (°C), *TMN* is minimum monthly temperature (°C), and *R*_*a*_ is extraterrestrial radiation (*MJm*^−2^*month*^−1^). In the ABCD model, soil water content is proportional to the evapotranspiration opportunity, and it exponentially decreases with the increases of the potential evapotranspiration rate (Eq. 6):

6 $$S{S}_{t}=Y{Y}_{t}\,exp(\frac{-PE{T}_{t}}{b})$$

Groundwater storage (*GG*, mm) and streamflow (or total runoff: *QQ*, mm) are calculated as functions of the available water and the evapotranspiration opportunity using Eq. 7 and Eq. 8, respectively:

7 $$G{G}_{t}=G{G}_{t-1}+c(W{W}_{t}-Y{Y}_{t})-dG{G}_{t}$$

8 $$Q{Q}_{t}=(1-c)(W{W}_{t}-Y{Y}_{t})+dG{G}_{t}$$

where *c* is a parameter that partitions water available for runoff (*WW*_*t*_−*YY*_*t*_) into direct runoff (*DR*, mm), $(1-c)(W{W}_{t}-Y{Y}_{t})$, and groundwater recharge, $c(W{W}_{t}-Y{Y}_{t})$. *d* is the groundwater residence time that is proportional to the baseflow recession constant, and *dGG*_*t*_ represents groundwater discharge (*GW*, mm). Evapotranspiration (*ET*, mm) is then regarded as the difference between precipitation and total runoff.

### Multi-parameter ensemble

The ABCD model prepared for each watershed was calibrated to the monthly streamflow measured at the outlets from 2008 to 2012. The calibration period was selected considering the availability of runoff observations to be used in parameter calibration. A sampling-based optimization algorithm, SCE-UA^76,101^, was used to explore the parameter space and to find sets of the five parameters, *a*, *b*, *c*, *d*, and *e* that provide acceptable model performance statistics during the calibration period. Monthly runoff hydrographs observed at the outlets of selected watersheds and simulated using the calibrated models are compared in Fig. S7 In the calibration, multiple parameter sets that satisfy the predefined performance requirements were identified as behavioral sets under the GLUE framework^57^. These behavioral sets are defined as “equally good” and “equally acceptable”^41^. To take subjectivity into account in the parameter uncertainty estimation, the combinations of an absolute threshold of the minimum NSE of 0.67 and the four different relative thresholds of the best 10%, 7.5%, 5%, and 2.5% were applied in identifying behavioral parameter sets out of those sampled in the calibration. It is worth noting that this study initially included 156 candidate watersheds for which USGS streamflow gage data are available within the Ohio River basin, and watersheds where the ABCD models did not meet the absolute performance criterion (NSE of at least 0.67) were excluded from this study. In Fig. S7, a parameter set that provided the highest NSE value was selected for each watershed.

### Quantification of uncertainty in multi-parameter and multi-GCM ensembles

**Figure 12:**
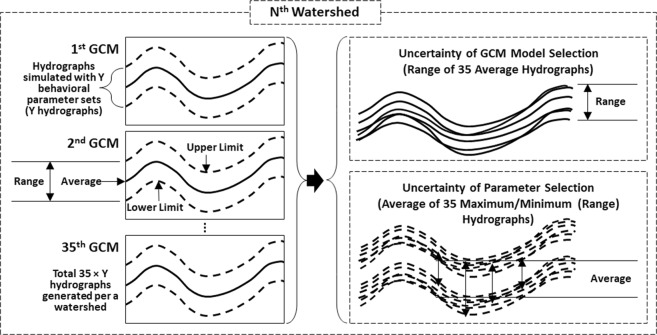
Processes for quantifying uncertainty in multi-GCM and multi-parameter ensembles. N varies from 1 to 61, i.e. the number of the Ohio River watersheds selected for this study; Y represents the number of behavioral parameter sets identified for each watershed and therefore varies by the watersheds.

The difference between maximum and minimum values (ranges) of projected climate variables (precipitation and temperature) and hydrological components (QQ, DR, SS, etc.) was calculated as a measure of the amount of uncertainty contained in ensemble predictions made using multiple GCMs and multiple (behavioral) parameter sets. Uncertainty in the multi-parameter ensembles was first quantified by calculating the ranges (the differences between upper and lower limits) of the monthly hydrographs simulated using the behavioral parameter sets that had been previously identified for each combination of GCMs and study watersheds (Fig. 12). Then, an average hydrograph of multi-parameter ensembles was derived for each GCM and study watershed combination, and the range of the variations in the average hydrographs across the GCMs for each watershed was regarded as the amount of uncertainty in the multi-GCM ensembles (Fig. 12). Thus, the uncertainty amount quantified for two different sources, multiple hydrological models (or parameter sets) and multiple GCM ensembles, is independent of each other, which allows a direct comparison of the two uncertainty quantities. The range is a statistical measure that has been commonly used to quantify uncertainty in hydrological modeling^102–104^. The range could show the maximum amount of uncertainty we could have from selecting GCMs and hydrologic model parameters in this study. Furthermore, the range could provide more straightforward and explicit quantification of uncertainty, compared to other statistical measures such as interquartile range and variance, as it is a direct measure of the spread of data.

The contribution of each GCM model to the GCM model selection uncertainty was quantified by comparing the uncertainty amount (ranges) in either the GCM ensemble predictions of the monthly climate variables or the hydrological components that were made with/without the use of each GCM (Eq. 9), as follows:

9 $${U}^{Q,m}(GC{M}_{x})={U}^{Q,m}(GC{M}_{\forall x\in S})-{U}^{Q,m}(GC{M}_{x\notin S})$$

where ${U}^{Q,m}(GC{M}_{x})$ is the uncertainty quantities in the GCM ensemble predictions made for either a climate variable or a hydrological component *Q* (e.g. PP and QQ) in the *m* month, which are solely attributed to *GCM*_*x*_; ${U}^{Q,m}(GC{M}_{\forall x\in S})$ is the total uncertainty in the GCM ensemble made for the *m* month; and ${U}^{Q,m}(GC{M}_{x\notin S})$ is the measured uncertainty in the GCM ensemble for which *GCM*_*x*_ is excluded. From the set theory of mathematics, *x* means an element (i.e. a climate model), *S* is a set of elements (i.e. a set of climate models), $\forall \,x\,\in \,S$ signifies all elements in *S*, and $x\,\notin \,S$ represents that an element *x* is not in *S*. Eq. 9 calculates the overall variation ranges (${U}^{Q,m}(GC{M}_{\forall x\in S})$) of climate variable and hydrological component projections made using all climate models ($\forall \,x\,\in \,S$) for the *m* month. Then, the equation subtracts the variation ranges (${U}^{Q}(GC{M}_{x\notin S})$) of the projections made excluding a specific climate model ($x\,\notin \,S$) from the overall variation ranges to quantify the uncertainty contribution of the specific model (*x*). In addition, the relationships between the quantities of uncertainty in the ensemble projections of the climate variables and hydrological components were then investigated to see which climate variable (precipitation, maximum and minimum temperatures) exerted the most significant influence on the hydrological prediction uncertainty.

## Supplementary information

Supplementary Information
