# Supplementary material for: Uncertainty in hydrological analysis of climate change: multi-parameter vs. multi-GCM ensemble predictions
Source: Sci Rep. 2019 Mar 21;9:4974. doi: 10.1038/s41598-019-41334-7 (PMC6428897; doi:10.1038/s41598-019-41334-7)
Supplement: Supplementary file 1 — Supplementary Information [file 41598_2019_41334_MOESM1_ESM.pdf]

Uncertainty in hydrological analysis of climate change: multi-parameter vs. multi-GCM ensemble predictions

Younggu Her[1], Seung-Hwan Yoo[2*], Jaepil Cho[3], Syewoon Hwang[4], Jaehak Jeong[5], and Chounghyun Seong[6]

[1] Department of Agricultural and Biological Engineering / Tropical Research and Education Center, Institute of Food and Agricultural Sciences, University of Florida, Homestead, Florida, United States

[2] Department of Rural and Bio-Systems Engineering, Chonnam National University, Gwangju, Republic of Korea

[3] Research Department, APEC Climate Center, Busan, Republic of Korea

[4] Department of Agricultural Engineering, Institute of Agriculture and Life Science, Gyeongsang National University, Jinju, Republic of Korea

[5] Department of Agricultural and Biological Engineering / Blackland Research and Extension Center, Texas A&M AgriLife Research, Texas A&M University, Temple, Texas, United States

[6] Bureau of Watershed Management & Modeling, St. Johns River Water Management District, Palatka, Florida, United States

[*] yoosh15@jnu.ac.kr

Author Contributions

Y.H. and S.Y. conceived the study. S.Y., J.C., and S.H. prepared the climate data. Y.H. and J.J. calibrated the hydrologic model, and Y.H., S.Y., and C.S. implemented the experiments. All authors analysed the results and reviewed the manuscript.

Competing Interests: The authors declare no competing interests.

Supplementary Information

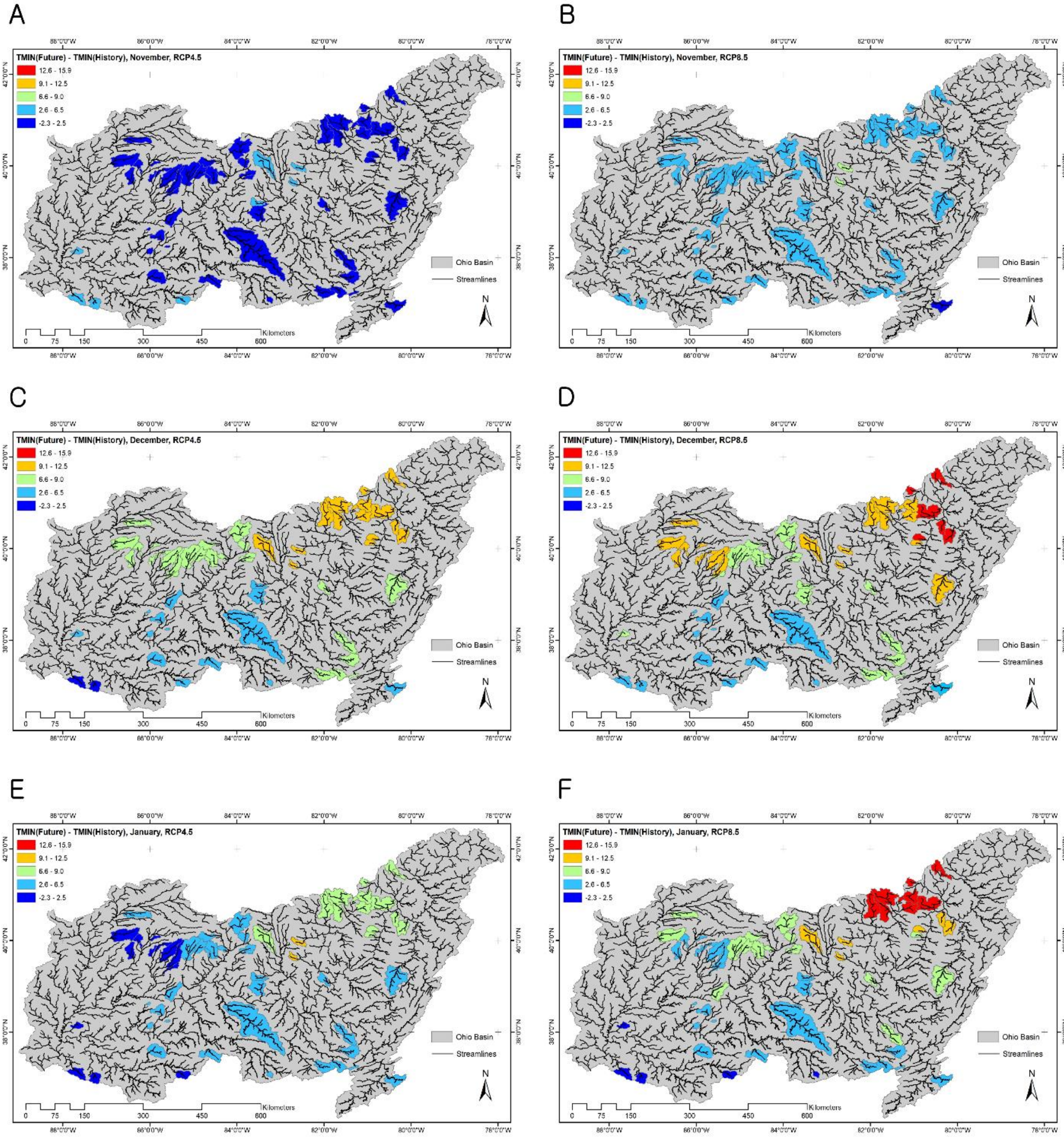

S1 Figure. Spatial distributions of the projected increases in the minimum temperature for selected months (November, December, and January).

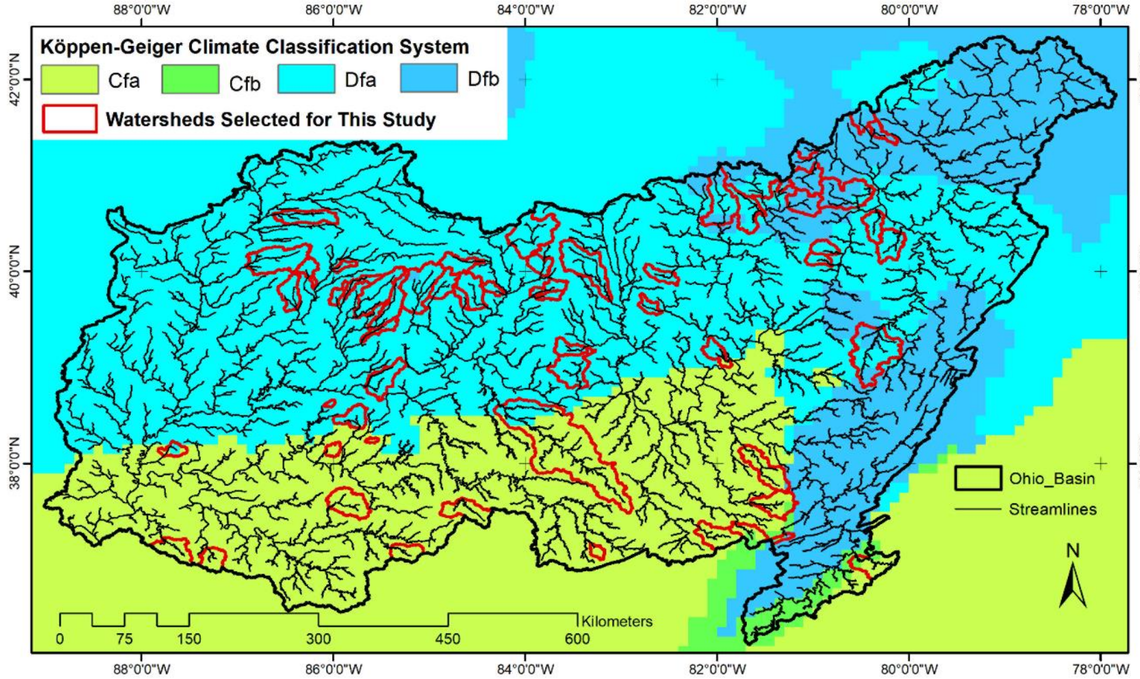

S2 Figure. Köppen-Geiger Climate Classifications of the Ohio River basin (Peel et al., 2007). Cfa: Temperate/Without Dry Season/Hot Summer, Cfb: Temperate/Without Dry Season/Warm Summer, Dfa: Cold/Without Dry Season/Hot Summer, and Dfb: Cold/Without Dry Season/Warm Summer.

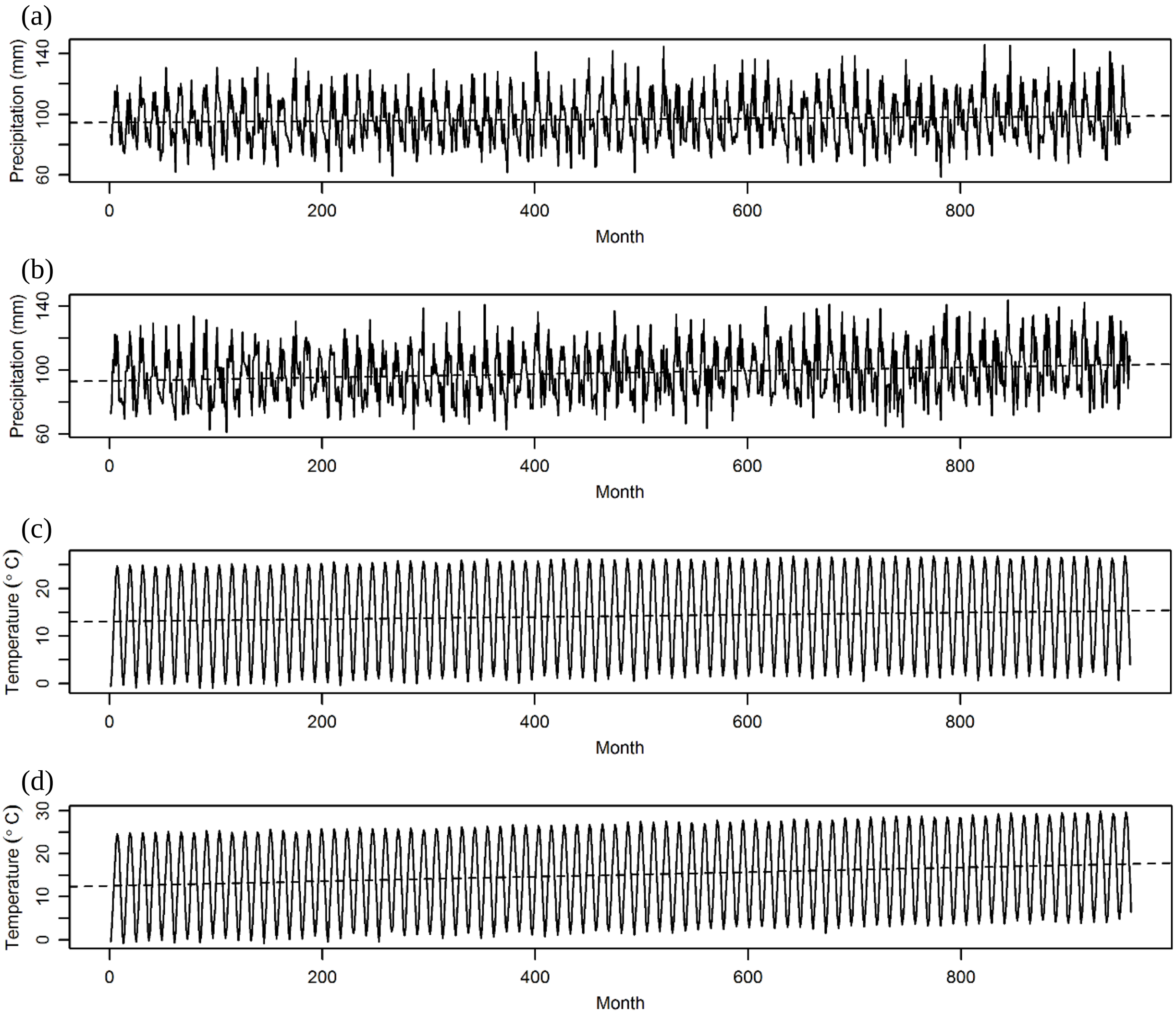

S3 Figure. Multi-GCM, multi-parameter, and multi-watershed ensemble projections (RCPs 4.5 and 8.5) of the overall average precipitation and temperature of the Ohio River watersheds from 2020 to 2099 (960 months). (a) RCP 4.5 – precipitation, (b) RCP 8.5 – precipitation, (c) RCP 4.5 – Temperature, and (d) RCP 8.5 – Temperature.

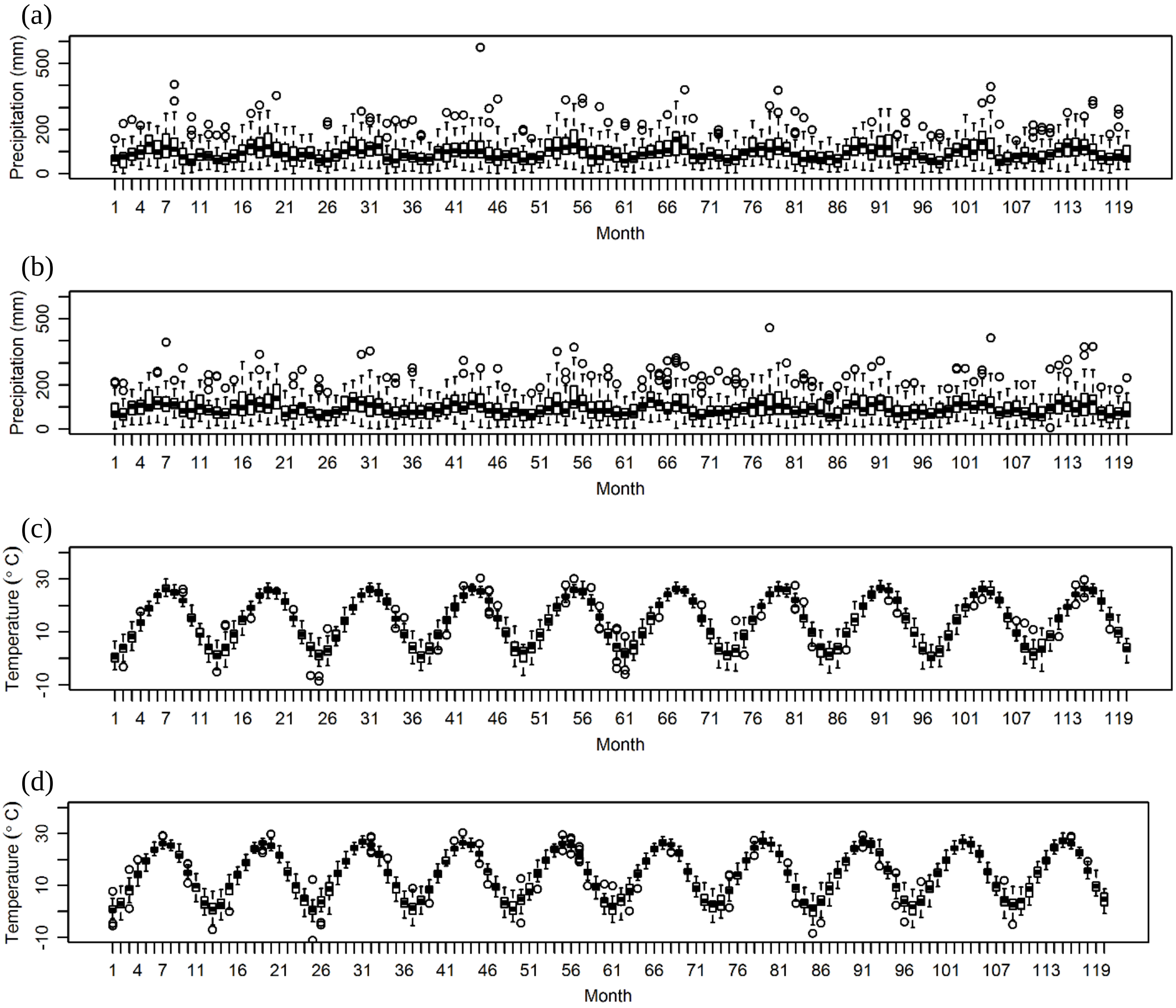

S4 Figure. Monthly variations of the precipitation and temperature projected by the 35 GCMs for the "03232500" (Rocky Fork near Barretts Mills, OH) watershed from 2030 to 2039 (120 months) under RCPs 4.5 and 8.5. (a) RCP 4.5 – precipitation, (b) RCP 8.5 – precipitation, (c) RCP 4.5 – temperature, and (d) RCP 8.5 – temperature.

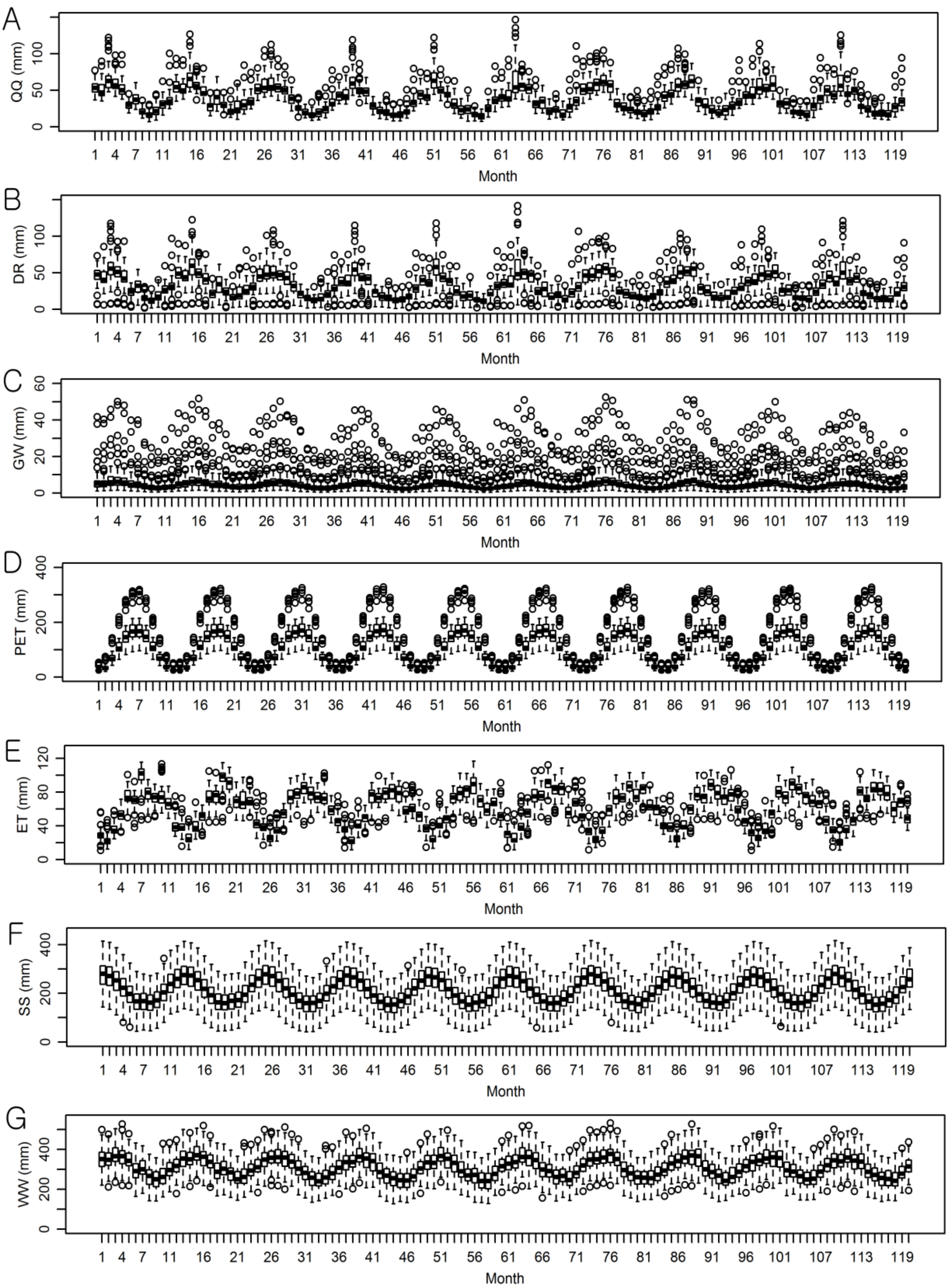

S5 Figure. Multi-parameter and multi-GCM ensemble projections for the hydrologic components (QQ, DR, GW, PET, ET, SS, and WA) of the study watersheds from 2030 to 2039 (120 months) under RCP 8.5.

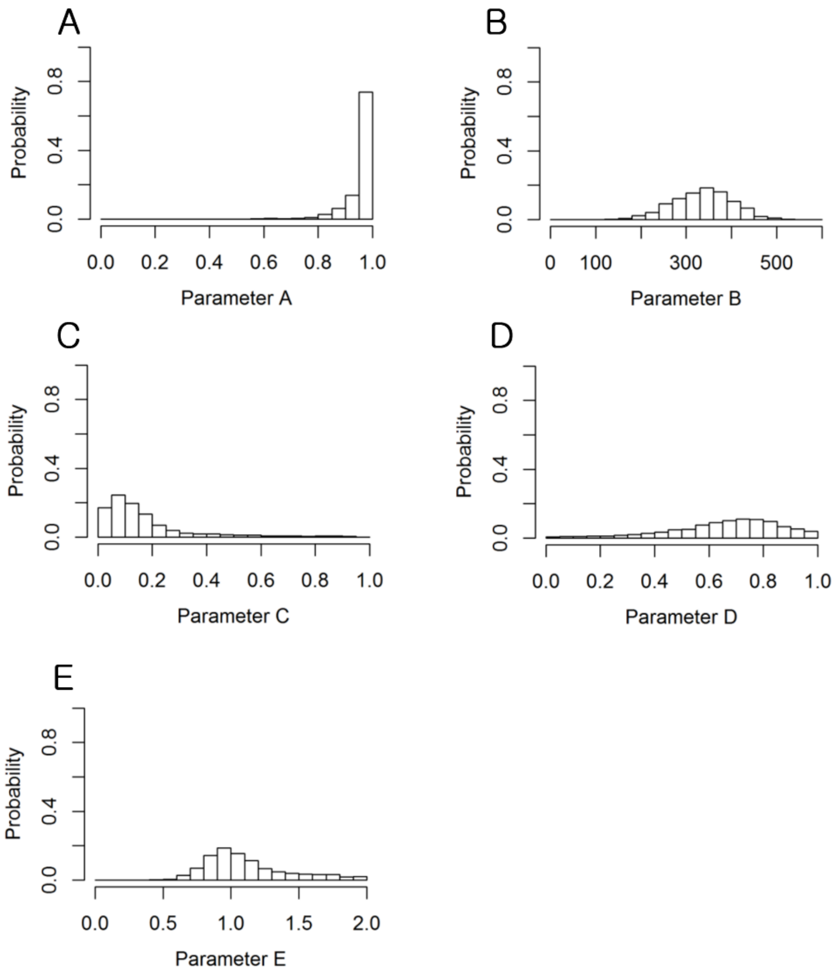

S6 Figure. Posterior distributions of the ABCD model parameters.

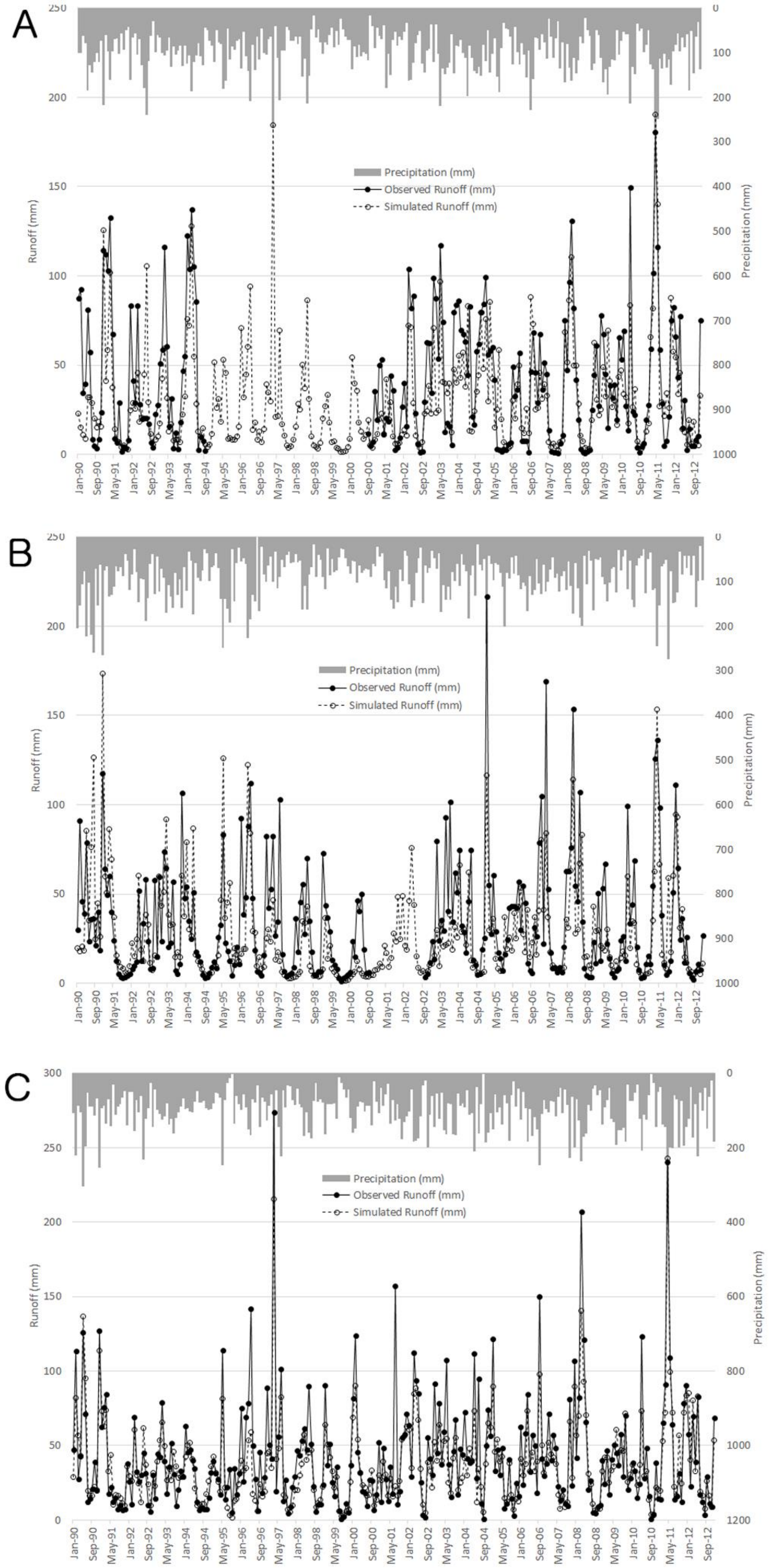

A
B
C
Precipitation (mm)
Observed Runoff (mm)
Simulated Runoff (mm)
Runoff (mm)
Precipitation (mm)

S7 Figure. Comparisons of runoff simulated and observed at the outlets of selected watersheds. (a) “03251500” (Licking River at McKinneysburg, KY; draining 6,024 km^2), (b) “03264000” (Greenville Creek near Bradford, OH; 500 km^2), and (c) “0329300” (M FK Beargrass CR at Old Cannons LN at Louisville; 49 km^2).
